# *US-SOMO* HPLC-SAXS module: dealing with capillary fouling and extraction of pure component patterns from poorly resolved SEC-SAXS data

**DOI:** 10.1107/S1600576716011201

**Published:** 2016-09-01

**Authors:** Emre Brookes, Patrice Vachette, Mattia Rocco, Javier Pérez

**Affiliations:** a Department of Biochemistry, University of Texas Health Science Center at San Antonio, 7703 Floyd Curl Drive, San Antonio, TX 78229-3901, USA; b Institute for Integrative Biology of the Cell (I2BC), CEA, CNRS, Université Paris-Sud, Université Paris-Saclay, Gif-sur-Yvette, F-91198, France; c Biopolimeri e Proteomica, IRCCS AOU San Martino-IST, Istituto Nazionale per la Ricerca sul Cancro, Largo R. Benzi 10, Genova, I-16132, Italy; d SWING Beamline, Synchrotron SOLEIL, L’Orme des Merisiers, BP48, Saint-Aubin, Gif-sur-Yvette, F-91192, France

**Keywords:** poorly resolved chromatographic peaks, asymmetric modified Gaussian decomposition, multi-resolution modeling, aldolase supramolecular complexes, *P*-value analysis, *CorMap* analysis, *US-SOMO* HPLC-SAXS module

## Abstract

Size-exclusion chromatography coupled with SAXS (small-angle X-ray scattering), often performed using a flow-through capillary, should allow direct collection of monodisperse sample data. However, capillary fouling issues and non-baseline-resolved peaks can hamper its efficacy. The *UltraScan* solution modeler (*US-SOMO*) HPLC-SAXS (high-performance liquid chromatography coupled with SAXS) module provides a comprehensive framework to analyze such data, starting with a simple linear baseline correction and symmetrical Gaussian decomposition tools [Brookes, Pérez, Cardinali, Profumo, Vachette & Rocco (2013 ▸). *J. Appl. Cryst.* **46**, 1823–1833]. In addition to several new features, substantial improvements to both routines have now been implemented, comprising the evaluation of outcomes by advanced statistical tools. The novel integral baseline-correction procedure is based on the more sound assumption that the effect of capillary fouling on scattering increases monotonically with the intensity scattered by the material within the X-ray beam. Overlapping peaks, often skewed because of sample interaction with the column matrix, can now be accurately decomposed using non-symmetrical modified Gaussian functions. As an example, the case of a polydisperse solution of aldolase is analyzed: from heavily convoluted peaks, individual SAXS profiles of tetramers, octamers and dodecamers are extracted and reliably modeled.

## Abstract 2

The *US-SOMO* HPLC-SAXS (high-performance liquid chromatography coupled with small-angle X-ray scattering) module is an advanced tool for the comprehensive analysis of SEC-SAXS (size-exclusion chromatography coupled with SAXS) data. It includes baseline and band-broadening correction routines, and Gaussian decomposition of overlapping skewed peaks into pure components.

## 1. Introduction   

Multi-resolution approaches for the structural characterization of complex macromolecular samples, such as in the presence of segmental/extended flexibility, or when supramolecular entities form in solution, are becoming increasingly used (see *e.g.* Ward *et al.*, 2013 ▸). Small-angle X-ray scattering (SAXS) is prominent in the multi-resolution toolbox, providing meso-resolution information over a wide range of sample sizes and conditions (Koch *et al.*, 2003 ▸; Putnam *et al.*, 2007 ▸; Svergun *et al.*, 2013 ▸). SAXS data result from an average over all species present in the solution sample; therefore separation/purification strategies are highly desirable to obtain interpretable results, especially when dealing with structure/shape analyses. Size-exclusion chromatography coupled on-line with SAXS detection (SEC-SAXS) is rapidly becoming the method of choice for collecting high-quality SAXS data on practically monodisperse samples (Pérez & Nishino, 2012 ▸; Kirby & Cowieson, 2014 ▸; Graewert & Svergun, 2013 ▸; Carter *et al.*, 2015 ▸). However, while this technique represents a major improvement over the traditional way of collecting data on samples pre-purified off-line, it is not problem free. As SEC-SAXS is often associated with a flow-through capillary sample holder, continuous exposure of the flowing sample to the intense X-ray beam can lead to capillary fouling, and closely related species or sample–column matrix interactions can result in overlapping and/or non-symmetrical peaks. While these two issues should preferably be dealt with at the experimental level (*e.g.* by reducing exposure times and/or adding radiation-damage protecting agents, or by changing the column type and/or length), this is not always possible. Moreover, while data quality assessment for ‘static’ samples is relatively easy, in SEC-SAXS it is not a straightforward task, since the signal changes continuously with elution time. Spurred by the need to analyze a particularly complex fibrinogen sample, a dedicated HPLC-SAXS (high-performance liquid chromatography coupled with SAXS) module was developed by Brookes *et al.* (2013 ▸) as a part of the small-angle scattering (SAS) section of the data analysis and simulation open source platform *UltraScan* solution modeler (*US-SOMO*; http://somo.uthscsa.edu/; Brookes, Demeler & Rocco, 2010 ▸; Brookes, Demeler *et al.*, 2010 ▸; Rocco & Brookes, 2014 ▸). In contrast with simpler programs that deal mainly with the automation of the repetitive tasks involved in analyzing the single different frames coming from a SEC-SAXS experiment (*e.g.* Shkumatov & Strelkov, 2015 ▸), the *US-SOMO* SAS and HPLC-SAXS modules were developed from their inception with the aim of providing advanced tools to deal with all aspects involved, from primary data treatment to the decomposition of unresolved components, and the comparison with model curves derived from high-resolution data. This last step is currently based on the embedded well known programs *Crysol* (Svergun *et al.*, 1995 ▸) and *Foxs* (Schneidman-Duhovny *et al.*, 2013 ▸).

**Figure 1:**
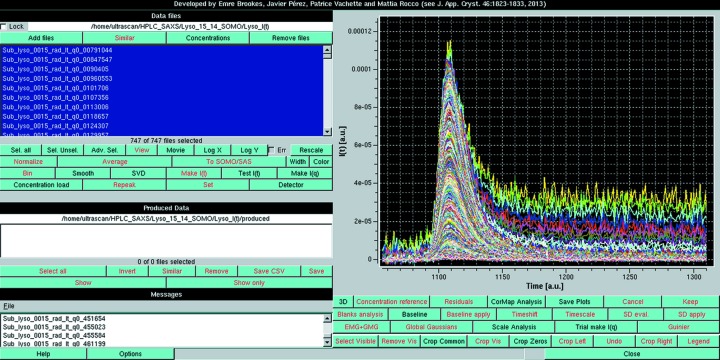
The main GUI of the *US-SOMO* HPLC-SAXS module. Shown are the *I_q_*(*t*) *versus t* chromatograms resulting from the conversion of a series of experimental *I_t_*(*q*) *versus q* frames collected on a lysozyme solution eluting from a SEC column (see the experimental procedures). The data were automatically trimmed to remove chromatograms containing only noise (see §S1 in the supporting information). The three lowermost *q* values were also manually discarded as they were judged to be unreliable, being too close to the instrument beam stop. At the program level, the black- and red-labeled buttons determine the operations allowed or not allowed at each stage, respectively.

The first key step in the HPLC-SAXS *US-SOMO* module is the conversion of the ensemble of *n* time frames (*t*), each containing a scattering intensity *I_t_*(*q*) as a function of the momentum transfer *q* [*q* = 4π sin(θ)/λ, with 2θ the scattering angle and λ the incident radiation wavelength] yielded directly by the SEC-SAXS experiment, into a series of *m I_q_*(*t*) *versus t* ‘chromatograms’ for each *q* value, where *m* is the number of different *q* values. Without any further analysis, this conversion already allows an immediate visual inspection of data quality such as capillary fouling issues when a few or several *I_q_*(*t*) chromatograms show intensity data not returning to the pre-peak elution (‘baseline’) value, a phenomenon especially evident in the low-*q* range (see Fig. 1 ▸). Furthermore, owing to the higher scattering intensity of higher molecular weight species, issues such as non-baseline-resolved peak separation can be better exposed in the *I_q_*(*t*) chromatograms than in the concentration profile usually associated with a SEC-SAXS experiment.

Some utilities for solving or at least alleviating the above-mentioned problems have been present since the first release of the *US-SOMO* HPLC-SAXS module (Brookes *et al.*, 2013 ▸). A linear baseline tool offers a possible correction of all *I_q_*(*t*) chromatograms. For non-baseline-resolved peaks, single-value decomposition (SVD) analysis of the data set can inform the choice of the minimal number of components (*i.e.* species) necessary to account for the data. Each species can then be associated with a function describing its elution profile. Since this profile in chromatography in general, and in SEC in particular, should in principle be well described by a series of symmetrical Gaussian functions (Delley, 1986 ▸), this was our initial choice (Brookes *et al.*, 2013 ▸). The Gaussian decomposition of the ensemble of *I_q_*(*t*) chromatograms into peak components is made possible through dedicated tools. The concentration signal (either UV–Vis or refractive index monitors are supported) can likewise be decomposed. Finally, from the *I_q_*(*t*) Gaussian-decomposed chromatograms, a series of *I_t_*(*q*) time frames for each baseline-corrected peak can be back-generated, which can then be further processed by the *US-SOMO* main SAS module. If the concentration signal is also available and has been processed, the relative concentration associated with each back-generated (decomposed) *I_t_*(*q*) frame, as well as the partial specific volume and the extinction coefficient (or d*n*/d*c*) of each species, can be carried over automatically. When not already done at the level of the beamline data-acquisition software, the data can then be put on an absolute scale using the reference *I*_0_ value of a standard scatterer, making it straightforward to derive the molecular weight (or mass/length or mass/area ratios) associated with each peak species.

We describe here important developments that have been made in the last *US-SOMO* HPLC-SAXS module release. Recognizing that the linear baseline-correction tool was not appropriate to account for capillary fouling but only for simpler cases such as drifting problems, a much more sound integral baseline-correction procedure has been implemented under the assumption that fouling accumulates proportionally with the intensity scattered by the sample while in the X-ray beam. Gaussian decomposition is no longer limited to symmetrical Gaussians; instead three types of skewed Gaussian functions are now present (see Di Marco & Bombi, 2001 ▸). This capability is similar to what is offered in commercial packages which, however, only operate on a single chromatogram at a time (*e.g. PeakFit*, Systat Software, San José, California, USA; http://www.sigmaplot.com). Moreover, a series of data processing and visualization tools operating on the *I_q_*(*t*) chromatograms [and partially on the original *I_t_*(*q*) data as well] have been added. These tools allow, for instance, the temporary back-generation of *I_t_*(*q*) frames and interactive estimation of the r.m.s. *z*-average square radius of gyration [〈*R*_g_^2^〉*_z_*]^1/2^ of the various species present. Importantly, advanced statistical tools based on the correlation map approach (*CorMap*; Franke *et al.*, 2015 ▸) have been implemented and can be employed whenever users are required to make choices or to evaluate results (although complete task automation would be highly desirable, we believe it prudent to postpone this task until a large number of user data sets from multiple beamlines are analyzed). A general restyling of the graphical user interface (GUI) has been carried out, thus simplifying operations, and additional utilities (*e.g.* the possibility of exporting data present in graphs to .csv type files) are available in the latest release of the SAS and HPLC-SAXS modules of *US-SOMO*.

The efficacy and usefulness of these new tools are first demonstrated with the adequate correction for capillary fouling in the SEC-SAXS analysis of a lysozyme sample. The tools are then applied to the extraction of pure individual *I_t_*(*q*) frames for higher-order complexes in the SEC-SAXS analysis of an aldolase sample, and their subsequent comparison with theoretical curves derived from high-resolution model structures.

## 2. Materials and methods   

### 2.1. Experimental and data processing   

All chemicals were reagent grade from Sigma–Aldrich (https://www.sigmaaldrich.com), and MilliQ water was used in the preparation of all the solutions. For the HPLC-SAXS analysis of lysozyme and aldolase, the buffers used were HEPES 50 m*M*, NaCl 100 m*M* pH 7, and Tris–HCl 50 m*M*, NaCl 100 m*M* pH 7.5, respectively. The lysozyme conditions were found to produce a high level of capillary fouling in an unrelated series of experiments, and were thus utilized on purpose in the context of the present work. No attempt to improve the experimental conditions was further pursued. Size-exclusion (SE)-HPLC was performed on a BioSec-3 (3 µm particle size, 300 Å pore size) 4.6 × 300 mm column (Agilent; http://www.chem.agilent.com). The Agilent chromatographic system on the SOLEIL synchrotron SWING beamline (David & Pérez, 2009 ▸) was operated at a flow rate of 0.2 ml min^−1^. The columns and the SAXS flow cell were maintained at 288 ± 0.1 K. Lyophilized hen egg white lysozyme (L-4919, Sigma–Aldrich) and rabbit muscle aldolase (A-2714, Sigma–Aldrich) were dissolved at nominal concentrations of ∼15 and ∼5 mg ml^−1^, respectively, in their respective elution buffers, and 5 µl samples were then injected in the SE column. Individual SAXS frames of 1 and 1.5 s, respectively, with a 1 s gap time between frames were collected at a sample-to-detector distance of ∼1.8 m, accessing a *q* range of 7 × 10^−3^ to 0.5 Å^−1^ (λ = 1.03 Å). All *I_t_*(*q*) frames were normalized to the intensity of the transmitted beam, radially averaged and background-subtracted using the local dedicated program *Foxtrot* (David & Pérez, 2009 ▸; freely available to academics upon request from the Xenocs company: foxtrot@xenocs.com). After conversion to *I_q_*(*t*) chromatograms and data processing in the *US-SOMO* HPLC-SAXS module, the back-generated *I_t_*(*q*) frames were put on an absolute scale when necessary using the scattering by water and then converted to units of g mol^−1^ within the *US-SOMO* SAS module. The aldolase extinction coefficient (*E* ^280^ = 0.877 ml mg^−1^ cm^−1^) was calculated from the composition by *PROMOLP* (Spotorno *et al.*, 1997*a* ▸,*b* ▸); its partial specific volume (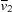 = 0.736 ml g^−1^) and the molecular weight of the tetramer (157 131 Da) were calculated from the crystallographic structure file by *US-SOMO*. Automated docking with SAXS profile restraints was performed with the *ClusPro 2.0* server (Comeau *et al.*, 2004 ▸; Kozakov *et al.*, 2006 ▸, 2013 ▸; http://cluspro.bu.edu/login.php). Final SAXS profiles for all the atomic scale models were calculated utilizing the *WAXSiS* server, which takes into account the scattering from explicit hydration-layer water molecules obtained from an all-atom molecular dynamics simulation with no adjustable parameter (Chen & Hub, 2014 ▸; Knight & Hub, 2015 ▸; http://waxsis.uni-goettingen.de/). Curves were generated up to *q*_max_ = 0.3 Å^−1^ and with a solvent electron density of 335 e nm^−3^. Molecular-model images were prepared with *UCSF Chimera 1.8.1* (Pettersen *et al.*, 2004 ▸; http://www.cgl.ucsf.edu/chimera/).

### 2.2. Software implementation and general program features   

The technical specifications of *US-SOMO* have been described previously (Brookes, Demeler & Rocco, 2010 ▸; Brookes, Demeler *et al.*, 2010 ▸; Brookes *et al.*, 2013 ▸). The software is primarily distributed as a binary GUI application for Linux, Mac OSX and Windows. Source code, written in C++ utilizing Qt (http://qt-project.org/), is freely available in subversion repositories as described on the *US-SOMO* wiki page (http://wiki.bcf2.uthscsa.edu/somo/). Registrations over­lap with the parent *UltraScan* package, which currently has over 1500 registered individual researchers and 53 registered laboratories worldwide.

Irrespective of the operating system used, the main *US-SOMO* program needs to be launched to access the HPLC-SAXS module. A full description of all the commands in this module can be found in the associated manual which is accessible by pressing the ‘Help’ button in the left-hand corner of the lower commands row of each window when running the program, and can be found directly at the URL http://somo.uthscsa.edu/manuals.php.

### 2.3. Theory   

#### 2.3.1. Integral baseline concept   

The integral baseline method is based upon the assumption that capillary fouling deposits are formed in proportion to the sample concentration while exposed to the beam, and that neither the buffer nor the instrumental background contribute to this effect. That deposition on the capillary does occur is clearly proven by the fact that a steady SAXS signal is maintained even after completion of the protein elution. Assuming further that the beam characteristics and detector response are constant throughout the duration of the experiment and the reference buffer’s signal has been correctly subtracted from the experimental data, then the remaining positive signal contains the sample’s scattering plus any capillary fouling. For a first approximation, we suppose that no ‘cleaning’ of the capillary takes place during the elution phase, that the capillary fouling is proportional to the sample’s scattering intensity while exposed to the beam, and that the proportionality coefficient is species independent. Here, ‘species’ refers to different aggregation states of a macromolecule (monomers, dimers *etc.*) or the presence of different macromolecular entities (*e.g.* ligand–receptor). While especially in the latter case this might be a rather strong assumption, it is a first approximation that could be further refined if new experimental evidence appears. Additionally, the possibility of using different coefficients for each species is already present in our implementation (see below). However, fine-tuning it might be not a straightforward task, and therefore we have for the moment restricted our analysis to the species-independent case.

If the data set *I*(*q*, *t*) with frames *T* = {*t*_1_, *t*_2_, …*t_n_*} has been correctly buffer-subtracted, then *I*(*q*, *t*) = 0 when only buffer is present and no fouling deposits have accumulated. To utilize our procedure, it is necessary to have a steady-state signal after all species have eluted and only buffer remains in the flowing solution. If this has not been achieved experimentally, it is difficult to proceed further. In the following, we will then assume a steady-state end signal. A robust procedure to evaluate whether the steady state has effectively been reached has been implemented (see §3.2).

Given *m* end frames of steady-state signal (*m* > 10 at least, but the longer this stretch the better), we define *t*_s1_ and *t*_s*m*_ as the beginning and ending frames of this region. Then, we can define the steady-state average as *I*_BL_(*q*), where BL indicates the final baseline:
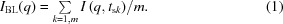
Now, if *I*_BL_(*q*) ≃ 0, then the signal has returned to a pure buffer condition and no correction is needed. If *I*_BL_(*q*) < 0, it means that net deposited material was removed from the cell, and this is contrary to our assumption. *I*_BL_(*q*) > 0 instead means that capillary fouling deposits were formed, which is the case considered from now on. We first define the unknown baseline correction for the capillary fouling deposits as *B*(*q*, *t*). Notice that *B*(*q*, *t*) should increase monotonically with *t* if deposits are only accumulated. Let *D*(*q*, *t*_*k*_) = *B*(*q*, *t_k_*) − *B*(*q*, *t*_*k*−1_) be the deposits accumulated from *t*_*k*−1_ to *t_k_*. From our hypothesis, we assume that *D*(*q*, *t_k_*) is proportional to *I*(*q*, *t*_*k*−1_) − *B*(*q*, *t*_*k*−1_), *i.e.* the signal above previously accumulated deposits. Specifically,
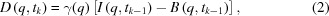
where γ(*q*) is a constant of proportionality. The goal of the integral baseline subtraction is to compute *B*(*q*, *t*) given *I*(*q*, *t*) [and given *I*_BL_(*q*), which is actually a subset average of *I*(*q*, *t*)].

The implemented integral baseline procedure computes *B*(*q*, *t*) iteratively. This follows naturally from the fact that we are only accumulating deposits as a proportion of the signal above the baseline and, as improved approximations for *B* are computed, we can compute an improved approximation of the signal from the species in solution. The algorithm proceeds as follows.

Note: *q* is fixed during a cycle, and this procedure is repeated for each *q*.

(i) Set the initial baseline to zero: *B*_0_(*q*, *t*) = 0.

(ii) Loop *i* = 0,…, maximum iterations.

(iii) Compute the total intensity above the baseline:

Let 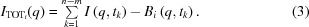



(iv) Compute 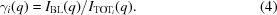



(v) If 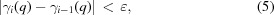
[where ∊ is the threshold value defined by the user (default value 0)] terminate early.

(vi) 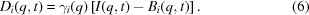



(vii) 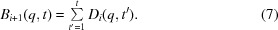



Note that *B*(*q*, *t*_0_) remains equal to zero throughout the algorithm. Physically, this represents the fact that no deposit due to irradiation is present at *t*_0_, since no sample exposure has occurred yet. It does not mean that the measured intensity at *t*_0_ is zero. Testing of the integral baseline algorithm was done with multiple simulated Gaussian data sets. For each Gaussian data set, a simulated experimental data set was created by adding γ multiplied by the intensity to simulate deposits and, additionally, random noise. The simulated experimental data were processed through the algorithm and correctly recovered the simulated Gaussian data.

#### 2.3.2. Non-symmetrical Gaussian functions   

In addition to the classical symmetrical Gaussian function
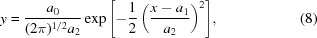
where *a*_0_, *a*_1_ and *a*_2_ are the area, center and width of the peak, respectively, the following non-symmetrical Gaussian functions (see Di Marco & Bombi, 2001 ▸) have also been implemented.

(i) The exponentially modified Gaussian (EMG)
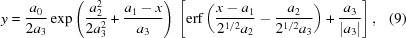
where *a*_0_, *a*_1_, *a*_2_ and *a*_3_ are the area, center, width and distortion of the peak, respectively.

(ii) The half-Gaussian modified Gaussian (GMG)
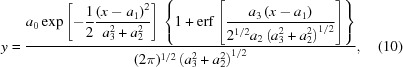
where, again, *a*_0_, *a*_1_, *a*_2_ and *a*_3_ are the area, center, width and distortion of the peak, respectively.

(iii) EMG + GMG
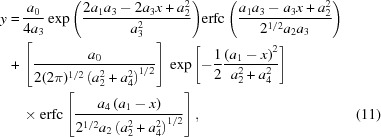
where *a*_0_, *a*_1_, *a*_2_, *a*_3_ and *a*_4_ are the area, center, width, first distortion and second distortion parameters of the peak, respectively.

#### 2.3.3. The *r*_σ_ multiplier used in the comparison of experimental and calculated *I_t_*(*q*) *versus q* data   

*r*_σ_ is utilized to produce a goodness-of-fit estimator χ *r*_σ_ that is independent of the global noise level observed with that particular data set. *r*_σ_ is defined as
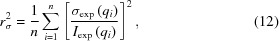
with σ_exp_(*q_i_*) the standard deviation (s.d.) associated with each *I*_exp_(*q_i_*) point and *n* the total number of points in the sum, while χ is given by the classical expression (Pearson, 1900 ▸)
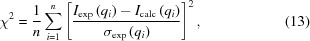
when comparing experimental and calculated intensities, and in all other instances when a χ estimator might be used.

#### 2.3.4. Defining a sound indicator of similarity using a *CorMap*-derived statistical analysis   

SEC-SAXS data analysis involves repeated curve comparisons and decisions regarding their similarity. This problem has recently been addressed by Franke *et al.* (2015 ▸) in a remarkable paper in which the authors proposed a novel goodness-of-fit test for assessing differences between one-dimensional data sets using only data-point correlations over the recorded *q* range or part of it, independently of error estimates, named *Correlation Map* (*CorMap* for short). We implemented a routine for the calculations described by Franke *et al.* (2015 ▸), in which we essentially perform pairwise comparisons of two scattering patterns. In this case, the probability of similarity between the two curves (two different frames or experimental and calculated intensities) may be quantified by evaluating the probability (*P* value) that the largest observed stretch of constant sign correlations occurs by chance. If the *P* value is less than a given threshold, the two curves are considered statistically different. We refer the reader to the original article for more details of the method.

In addition, we were then faced with the multiple testing problem: when testing hypotheses (here, comparing two curves), incorrect rejection of the null hypothesis (here, the identity of the two curves) is more likely to occur (type I error) when one considers multiple pairwise comparisons within a large set of curves (Miller, 1981 ▸). A simple way to account for this statistical effect has been proposed by Bonferroni (see Dunn, 1961 ▸), in which the acceptance threshold for each *P* value is divided by *m*, the total number of pairwise comparisons made. This adjustment appears to be permissive in some cases and thus prone to favor the null hypothesis, which would erroneously consider two curves that exhibit genuine differences to be identical (type II error). We chose to use the variant known as Holm–Bonferroni (Holm, 1979 ▸), which has a greater statistical power than the original Bonferroni adjustment (see supporting information for details) but still cannot be totally free from the aforementioned permissiveness. To assess the consequences of the bias brought by the multiple testing adjustment, we analyze in parallel the distribution of *P* values, as defined in *CorMap*, derived from all pairwise comparisons in a given data set of interest and compare it with that of a reference data set comprising only buffer frames. These buffer frames only differ from each other for purely statistical reasons or from uncontrolled biases due to the beamline setup. Their global level of similarity thus provides an internal reference with which to compare further data sets. The similarity test is performed without the Holm–Bonferroni (HB) adjustment, using stringent conditions to avoid considering as identical curves exhibiting genuine differences. In this case, the lack of a multiple testing adjustment is not an issue, since conclusions are drawn from the comparison of two identical analyses.

Regardless of the method used, we present the results in a synthetic way by plotting a square matrix in which the dot (*i*, *j*) contains the respective pairwise *P* value represented using a three-color code [the same as used in the *CorMap* implementation within the *PrimusQt* software (*Atsas* package version 2.7.1)]: green for *P* ≥ 0.05, yellow for 0.01 ≤ *P* < 0.05 and red for *P* < 0.01 when no multiple testing correction is applied. The same color code is utilized using the adjusted acceptance threshold when applying the HB procedure (see supporting information for the definition of the threshold). This pairwise *P*-value map is first analyzed in terms of the distribution of values between the three classes, with an emphasis on the percentage of red dots. In our unadjusted comparative approach, this is complemented by an evaluation of the average red cluster size, defined as maximal groupings of horizontally and/or vertically adjacent ‘red’ dots.

After careful examination of several indicators derived from the pairwise *P*-value map analysis, the average red cluster size was chosen as the most reliable one to determine the global similarity between all frames of the considered subset. However, we have observed that red *P* values are too few for clusters to be present when the HB adjustment is applied. In this case, the percentage of red pairs is used as the indicator. Conclusions are reached regarding the global similarity within the data set of interest from the comparison of the resulting distributions obtained for that data set and for the reference set.

We also provide a *q*_max_ cutoff for all *P*-value comparisons, which is 0.05 Å^−1^ by default but can be changed by the user. The primary purpose of this cutoff is to reduce the number of points compared in the region of greatest signal information and thereby increase the sensitivity of the probability test.

## 3. Results   

### 3.1. Pairwise *P*-value analysis of buffer frames   

The pairwise *P*-value approach without a multiple testing adjustment was used to analyze 89 buffer frames derived from the lysozyme SEC-SAXS experiments. As shown in the top panel of Fig. S1 in the supporting information, this approach resulted in more than 30% pairwise *P* values smaller than 0.01 (red squares). This was quite unexpected for a set of buffer frames assumed to be identical, suggesting that the corresponding two frames exhibit statistically significant differences. This could be attributed to the multiple testing effect which increases the rate of type I error. Indeed, applying the HB adjustment reveals 0.2% comparisons with a *P* value below the threshold (supplementary Fig. S2, top panel), a low value compatible with the sole presence of random noise between curves. However, we soon realized that this could be directly associated with the characteristics of both the beam cross section (0.4 × 0.15 mm FWHM) and the detector pixel size (0.17 × 0.17 mm) on the SOLEIL SWING beamline, which cause strong correlations between adjacent pixels. Indeed, performing the same analysis without a multiple testing adjustment on the same data set using every other *q* value yields hardly more than 2% of *P* values below 0.01, almost all of them isolated occurrences with no more than five red clusters of size two and no larger size cluster out of 3916 comparisons (supplementary Fig. S1, bottom panel). Although higher than the results of the HB adjustment, this remains close to what would be expected from the analysis of a set of identical curves. Finally, the combination of both corrections, in which we analyze the same data sampled every other *q* value using the HB adjustment, yields a completely green map with no significantly low *P* value (supplementary Fig. S2, bottom panel; see the dedicated §S2 in the supporting information for details).

We are facing here the case where an unexpected effect can be quite rightly attributed to both a physical and a statistical cause, each of which can practically fully account for it. Most likely, both contribute to the final result, but there is no totally objective way to disentangle one from the other. Therefore, the program offers both options for a more thorough evaluation of specific cases. Regarding the physical contribution, the pairwise *P*-value analysis without a multiple testing adjustment has thus clearly revealed the existence of local strong correlations. This would not be observed to such an extent were beam dimensions and pixel size more closely matched. On the basis of these results, we suggest that a preliminary check on an ensemble of buffer frames should first be performed before any pairwise *P*-value analyses on data sets, allowing an informed choice of the conditions under which these analyses should be conducted. Actually, it is most likely to be a one-off check on a given instrument followed by the appropriate selection of the pairwise *P*-value analysis parameters (*e.g.* using one every *n* *q* values).

If we strictly follow what is mentioned in the methods section, the level where a set of sample frames can be considered identical should be taken from the degree of similarity obtained from the ensemble of buffer frames, whether the multiple testing adjustment is introduced or not. However, a reference is, in principle, not required for a multiple testing correction. Supporting this statement, we have observed that the fraction of red *P* values with multiple testing on buffer frames is quite low (0.2%). Therefore, when adjusting for multiple testing effects, we deem a fraction of red *P* values from the sample frame data set below 1% as acceptable. In case of doubt, a comparison with the reference data set can always be performed.

### 3.2. Checking the baseline for capillary fouling evidence, and integral baseline correction   

Capillary fouling may occur when elution from a chromatography column is directly coupled to the SAXS measuring cell. This can already be detected from a visual inspection after an *I_t_*(*q*) to *I_q_*(*t*) transposition, as in the case of the lysozyme data set shown in Fig. 1 ▸, where a large number of *I_q_*(*t*) chromatograms do not return to baseline values after peak elution. However, a more rigorous procedure to detect the extent of capillary fouling and the need for corrective action has been implemented within the *US-SOMO* HPLC-SAXS ‘Baseline’ utility.

**Figure 2:**
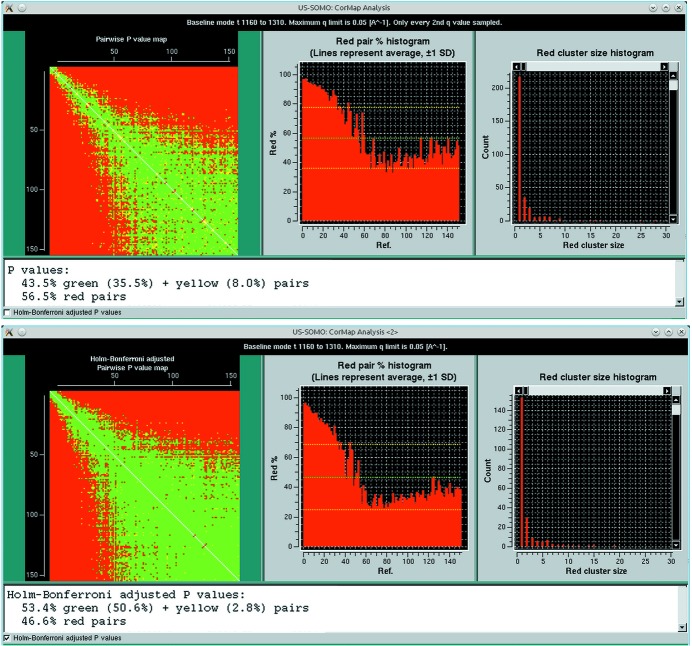
The results of the ‘Find best region’ pairwise *P*-value analysis in Baseline mode for the lysozyme data of Fig. S3 in the supporting information. The start and end frames were 1160 and 1310, with a 20-frame sliding window size. (Top panel) With one-every-second *q*-value sampling and no Holm–Bonferroni adjustment. (Bottom panel) Without sampling and with the Holm–Bonferroni adjustment. In both panels, the red cluster-size histogram window is enlarged to show the data relevant for the baseline definition.

**Figure 3:**
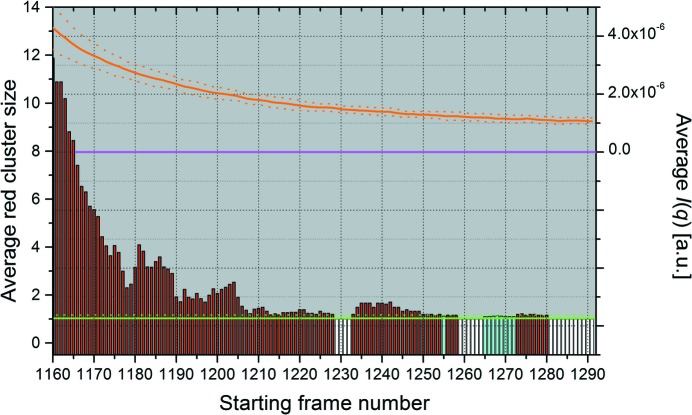
Graphical representation of the ‘Find best region’ pop-up panel derived from the pairwise *P*-value analysis with Blanks comparison of the lysozyme data of Fig. 2 ▸ and Fig. S3 in the supporting information (see Fig. S4 in the supporting information for a screenshot of the actual panel). On the left *y* axis, red bars indicate the average red cluster size for the chosen window size (here 20 frames) of the actual chromatogram as a function of each starting frame number, compared with the average Blanks values + 1 s.d. (green solid and dotted horizontal lines, respectively) computed for all possible windows of the same number of frames; cyan and white bars represent windows with an average within +1 s.d. of the Blanks, with the white bars being the lowest (equal) averages recorded. On the right *y* axis, the orange lines indicate the average integrated intensity ± 1 s.d. for each of the 20-frame windows analyzed on the actual chromatogram, as a function of each starting frame number; the magenta horizontal line indicates the zero expected intensity if no capillary fouling or other phenomena such as drifting have occurred.

As shown in supplementary Fig. S1, we begin with the preliminary pairwise *P*-value analysis of a set of SAXS frames collected on the buffer eluting from the chromatography column well before its void volume, typically the same frames that were then averaged and subtracted from all other experimental frames. This will constitute the reference data set (see §3.1). Thereafter, the first step in the baseline analysis is to determine a constant final baseline region. To that end, the pairwise *P*-value analysis of the data set of interest is performed over a window of typically 20 frames which slides over a predetermined time range, initialized as shown in supplementary Fig. S3. The first indicator is the pairwise *P*-value results, as summarized by the time evolution of average red cluster size over the specified window (Fig. 2 ▸, top panel) compared with those of the previously analyzed buffer average red cluster size over the same window. This indicator determines if a stable baseline region, with ‘identical’ frames, has been attained. The second indicator is the cumulative intensity over the *q* range used (*q* ≤ 0.05 Å^−1^ by default), averaged over all frames within the sliding window. This second indicator is computed to estimate the temporal stability of the signal and whether it returned to zero or not. Both indicators are plotted in a pop-up panel, whose results are summarized in Fig. 3 ▸ (see also supplementary Fig. S4, top). Additionally, we examine whether the minimum cumulative intensity over all windows is greater than zero or not at any point, and accordingly a suggestion is made regarding the possibility of applying an integral baseline correction. The examination of both indicators in Fig. 3 ▸ guides the choice of the frame window for the estimate of the final residual intensity at each *q* value to be used for fouling correction. In the case shown in Fig. 3 ▸, ‘flat’ regions are present in two or three separate zones according to the red cluster size indicator, but the examination of the average cumulative intensity appears to be a more stringent test and indicates that a stable baseline only forms at the end of the available frames, with a level clearly above zero confirming the need for correction.

We also performed the pairwise *P*-value analysis of the data set of interest utilizing the HB multiple testing adjustment, using all *q* values (Fig. 2 ▸, bottom panel). This is also complemented by our second indicator, the calculation of the cumulative intensity as explained above. The results are shown in supplementary Fig. S4 (bottom). Here again, the latter indicator is more stringent and leads to the same final choice for the final steady-state region to be used for baseline correction.

If an integral baseline correction is required, it can now be applied. The integral baseline procedure is based upon the assumption that capillary fouling deposits are formed in proportion to the scattered intensity by molecules in solution while exposed to the beam, and that neither the buffer nor the instrumental background contributes to this effect. A simplifying assumption considers that the proportionality coefficient is species independent within a given elution experiment. The implemented integral baseline procedure computes the correction iteratively following an algorithm detailed in §2.3.1.

Before applying the integral baseline procedure to all selected *I_q_*(*t*) chromatograms, the effects can be visualized on one chromatogram at a time. Shown in supplementary Fig. S5 is an original *I_q_*(*t*) curve at *q* = 0.0090 Å^−1^ (green), compared with the baseline-subtracted curve (dark orange) and with the five baseline curves produced by the iterative integral baseline subtraction procedure (from purple to light green; only four are visible because convergence in this case is reached by the fourth iteration). The original chromatogram is subjected to Gaussian smoothing (superimposed, blue) before the integral baseline computation, but the baseline subtraction is then applied to the original chromatogram. This smoothing procedure (over seven points by default) was introduced to avoid problems with large oscillations of the original data around the computed baseline iterations, which can be troublesome at low *q* values.

**Figure 4:**
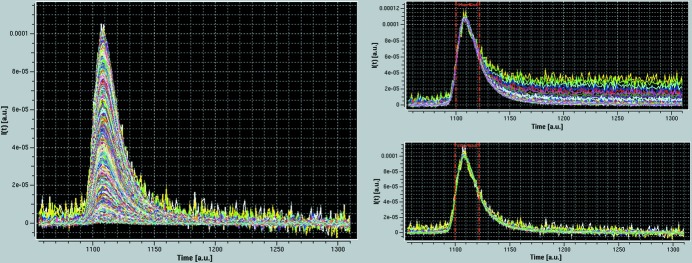
(Left-hand panel) The lysozyme SEC-SAXS chromatograms of Fig. 1 ▸ after integral baseline correction. (Top right-hand panel) A subset of the original *I_q_*(*t*) data from *q* = 0.00791 Å^−1^ to *q* = 0.05029 Å^−1^, superimposed and scaled to the maximum value in the frame interval 1100–1122. (Bottom right-hand panel) The same chromatograms scaled after integral baseline correction.

A final set of integral baseline-subtracted *I_q_*(*t*) chromatograms is shown in Fig. 4 ▸ (left-hand panel). Note that the integral baseline subtraction procedure always performs a test to verify that the integral is not negative, which would lead to an addition of signal rather than a subtraction. In this case, essentially encountered at larger *q* values in regions of vanishing signal, the integral baseline is not subtracted, a warning appears in the message window, and ‘0s’ is added to the resulting filename.

**Figure 5:**
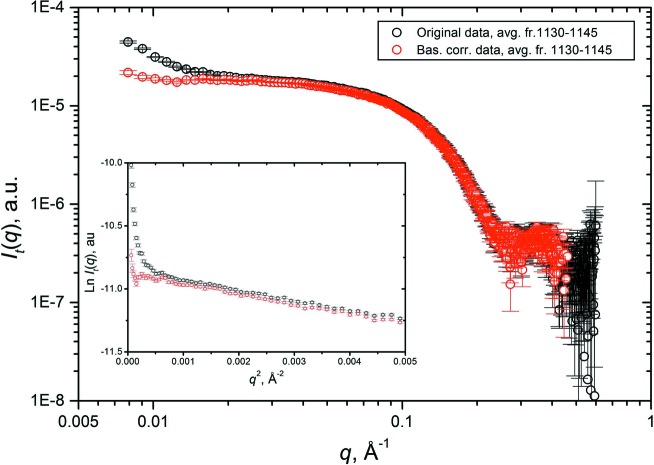
Comparison of original (black circles) and baseline-corrected (red circles) *I_t_*(*q*) averaged lysozyme SEC-SAXS frames 1130–1145. (Main panel) A log–log plot of scaled intensities. (Inset) A Guinier plot. In both panels, only one out of every two points is shown for clarity.

The dramatic effect of the integral baseline correction can be appreciated in the right-hand panels of Fig. 4 ▸, where the same subset of lysozyme *I_q_*(*t*) SEC-SAXS chromatograms ranging from *q* = 0.00791 to *q* = 0.05029 Å^−1^ are scaled on each other in a frame interval corresponding to the half-height of the peak, before and after baseline subtraction. The fact that the right-hand sides of the peaks are nicely superimposed after baseline subtraction validates *a posteriori* the procedure used to build the baseline, since for a single species the elution peaks at different *q* values should be strictly proportional to each other. A second check of the baseline correctness can be performed after back-generation of the *I_t_*(*q*) frames from the baseline-corrected *I_q_*(*t*) chromatograms. In Fig. 5 ▸, averages of frames 1130–1145, corresponding to a low-intensity zone on the descending side of the lysozyme peak, are shown, before (black circles) and after (red circles) baseline correction. Both the log–log plot of the scaled intensity (Fig. 5 ▸, main panel) and the Guinier plot (inset) evidence how the baseline-correction procedure has almost completely restored proper behavior in the low-*q* region.

### 3.3. Non-symmetrical Gaussian decomposition of overlapping peaks   

The second major improvement present in the current release of the *US-SOMO* HPLC-SAXS module is the possibility of using non-symmetrical Gaussian functions to decompose non-baseline-resolved peaks. We have implemented exponentially modified Gaussian (EMG) and half-Gaussian modified Gaussian (GMG) functions and a combination of the two (EMG + GMG) (see *Theory*, §2.3.2). Additional statistical tools have also been implemented to aid in judging the quality of the decompositions. This is particularly relevant since these are processes that are difficult to automate fully, and thus require direct user interaction at several steps.

**Figure 6:**
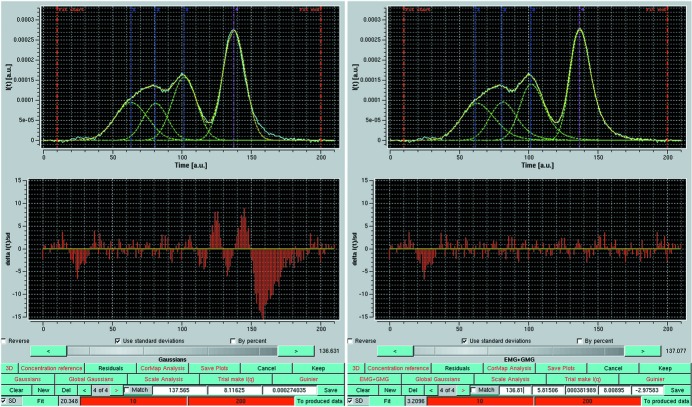
(Top left-hand panel) A single low-*q* *I_q_*(*t*) *versus t* chromatogram from the aldolase SEC-SAXS data set after fitting with four symmetrical Gaussian functions. (Top right-hand panel) The same chromatogram after Gaussian decomposition with four EMG + GMG non-symmetrical Gaussian functions. In both panels are shown the original curve (solid cyan line) superimposed with the reconstruction (yellow dashed line) and the four independent Gaussian functions (green dashed curves). (Bottom panels) The respective reduced residuals.

In Fig. 6 ▸ (top-left panel), a single low-*q I_q_*(*t*) chromatogram from a rabbit muscle aldolase SE-HPLC-SAXS data set (see *Materials and methods*, §2.1) is shown (cyan curve), together with the result of a Gaussian decomposition (yellow dashed curve) utilizing four symmetrical Gaussian functions [equation (8), green dashed curves; the center of each Gaussian is indicated by a vertical blue or magenta dashed line]. The number of Gaussians used was inferred from a preliminary SVD analysis (data not shown); the small ‘peak’ eluting before the major ones, only detected at low *q* values, corresponds to a very small amount of large oligomers and has not been included in the subsequent analysis. As can also be judged by the reduced residuals shown in the bottom-left panel, the fit is far from satisfactory, especially under the center and the falling edge of the highest peak. The data were then fitted again using EMG + GMG functions [equation (11)], which allow for distortions to be taken into account on both the rising and falling edges of each peak. The much improved results are shown in Fig. 6 ▸ (right-hand panels).

The non-symmetrical Gaussian behavior is probably due to interactions of the eluting sample with the column matrix. Since SEC-SAXS experiments are most often performed on samples containing different states of the same substance (*e.g.* monomers, oligomers and higher-order aggregates), it is reasonable to assume that the mode of interaction will be common to all species. Therefore, by default, the distortions are kept equal for each modified Gaussian peak [mG-Pk(*i*)] during the fitting phase. If there is evidence or reason to suppose that different peaks have different interaction modes with the column matrix, this constraint can be released.

The parameters associated with this set of EMG + GMG functions optimized on the single initially chosen *I_q_*(*t*) chromatogram are then accepted and used to initialize a global fit on a (large) subset of the available *I_q_*(*t*) chromatograms (in this example, one out of every four from *q* = 0.0103 Å^−1^ to *q* = 0.1103 Å^−1^). In this first initialization step, widths, centers and distortions for each peak ‘family’ in all the selected chromatograms are kept fixed and only the amplitudes are adjusted. The actual global fit is subsequently performed in a user-controlled way: each parameter can be either freely fitted or constrained to remain within an allowed range, but being common to all fitted chromatograms. Importantly, by default the distortions are, in addition, optimized to be also common to all modified Gaussians.

Once the results of the global fit are accepted, all chromatograms can then be selected, and all amplitudes are fitted for all selected chromatograms while using all the common width, center and distortion values resulting from the previous global fit operation (not shown). At the end of the operations, all EMG + GMG parameters can be saved into a file for later retrieval.

**Figure 7:**
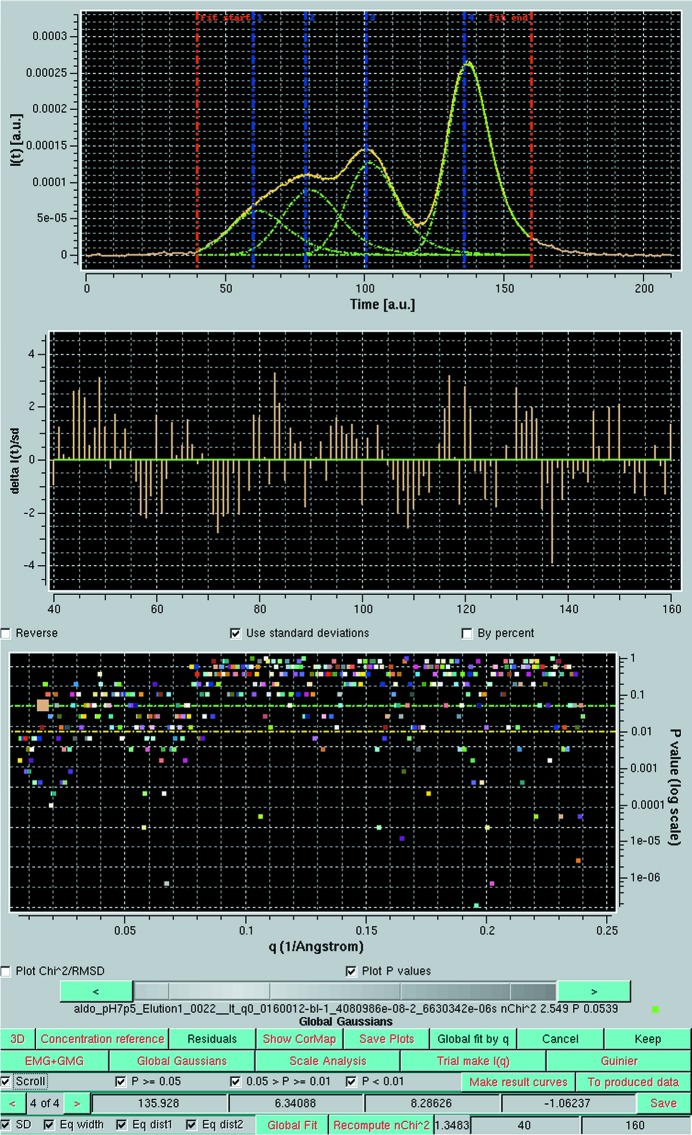
(Top panel) *I_q_*(*t*) *versus t* chromatogram for *q* = 0.01600 Å^−1^ (khaki line) together with the EMG + GMG fit (yellow line), with the individual EMG + GMG Gaussians shown as green lines; the red vertical lines indicate the limits for the goodness-of-fit evaluation. (Middle panel) The reduced residuals. (Bottom panel) The ‘Global fit by q’ plot, showing only the pairwise *P* values as a function of *q*; the currently scroll-selected chromatogram is indicated by the enlarged symbol. The horizontal yellow and green lines indicate the cut-off values for 0.05 > *P* ≥ 0.01 and for *P* ≥ 0.05, respectively.

An in-depth analysis of the fitting results can be done at each step using the ‘Global fit by q’ and ‘Scroll’ modes. The ‘Global fit by q’ presents the plots of two goodness-of-fit indicators as a function of *q*: χ^2^, and the *P* value derived from a pairwise analysis between each chromatogram *I_q_*(*t*) and the corresponding fit using modified Gaussians. The ‘Scroll’ mode allows the user to visualize each *I_q_*(*t*) chromatogram pair and associated reduced residuals, with its χ^2^ and the *P* value highlighted in the ‘Global fit by q’ plot. In Fig. 7 ▸ (top panel), a single chromatogram at *q* = 0.01600 Å^−1^ is shown, together with the corresponding fit (the individual Gaussians are also shown), with the reduced residuals reported in the middle panel. The bottom panel presents only the *P* values for all chromatogram fits, with the current pair highlighted. The horizontal dotted lines indicate the cut-off values used for the definition of the three *P*-value classes. As can be seen in Fig. 7 ▸, the great majority of all fits at all *q* values have acceptable and good *P* values (above the yellow and green dotted lines), with the poor values being quite scattered. Note that, for this analysis, we have restricted the limits of the Gaussians’ evaluation (red lines in the top plot) to avoid including the very noisy regions at the beginning and end of the chromatograms. The fit to the chromatogram displayed in Fig. 7 ▸ has a high *P* value and exhibits low-amplitude reduced residuals. The examination of a poorly fitted chromatogram with *P* < 0.01, such as the one displayed in supplementary Figs. S6 and S7, illustrates a typical situation giving rise to low *P* values: the longest stretch of residuals having the same sign occurs in the trough between EMG + GMG peaks 3 and 4, where the fit is mostly slightly above the original data. Given also that most residuals are within ±2 s.d., this should hardly be of concern, especially considering that the frames that will be subsequently averaged for final analysis will mostly come from the top of the peaks, where the fit is more robust.

We have also revisited the way in which uncertainties are propagated after the (modified) Gaussian decomposition process [in the remainder of the *Results* section, we will refer generally to ‘Gaussian(s)’ for both normal and modified Gaussian functions, unless specifically stated]. The experimental uncertainties associated with every *I_q_*(*t*) point in the original chromatograms are first reassigned equally to the same *I_q_*(*t*) points in all derived *I_q_*(*t*) Gaussians. When back-generating *I_t_*(*q*) frames, it is also possible to add to each *I_q_*(*t*) original uncertainty a fraction of the calculated discrepancy between the chromatogram and its Gaussian fit, estimated from the relative intensity of each Gaussian contributing to that point. Each final uncertainty in the decomposed *I_t_*(*q*) frames is therefore computed as the root of the sum of the squares of the original and fit-derived uncertainties.

### 3.4. Analysis of the Gaussian decomposition using the new ‘Test I(q)’ mode   

The results of the non-symmetrical Gaussian decomposition can be compared with the unprocessed chromatograms using the ‘Guinier’ approximation available in the ‘Test I(q)’ mode. The program first back-calculates a set of *I_t_*(*q*) frames for the time/frame interval selected, plots them according to the Guinier representation {ln[*I_t_*(*q*)] *versus q* ^2^} and applies a linear fit, optionally also showing the fit residuals. The *q* range for the Guinier linear regressions can be adjusted manually, or the upper limit (*q*_max_) can be set automatically by choosing a limiting value for *q*_max_ *R_g_*. As a further utility, the whole data set can be scrolled through and each ln[*I_t_*(*q*)] *versus q* ^2^ fit visualized separately. A summary of the Guinier analysis results is shown, together with a calculation of the approximate molecular mass using the Rambo & Tainer (2013 ▸) approach, *M*_w_[RT], which also involves the calculation of the ratio between an integral of the SAXS curve and the Guinier-extrapolated *I*(0). On the basis of extensive trials (M. Brennich, ESRF, Grenoble, France, personal communication), the data included in the integral are limited to *q*_max_ = 0.2 Å^−1^. If the available *q* range is <0.2 Å^−1^, or if the default values are changed, warnings are issued alerting of the potential unreliability of the molecular mass estimates.

**Figure 8:**
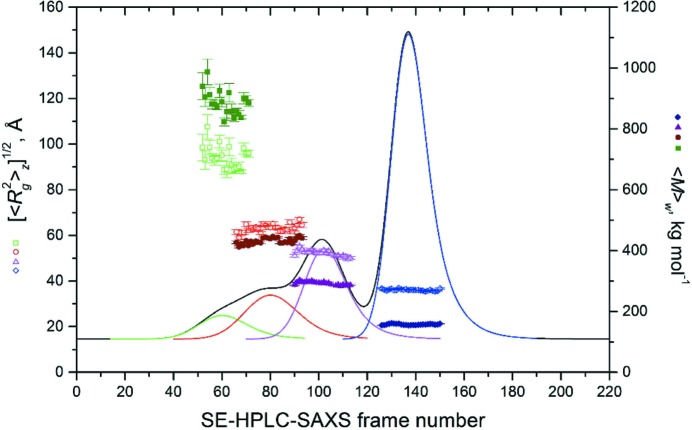
Plots of the [〈*R*_g_^2^〉*_z_*]^1/2^ (open symbols, light colors, left-hand scale) and 〈*M*〉_w_ (solid symbols, dark colors, right-hand scale) values obtained from Guinier analyses. Selected *I_t_*(*q*) *versus q* frames were back-generated after the EMG + GMG decomposition of the SE-HPLC-SAXS aldolase data set. All frames were normalized to protein concentration following concentration chromatogram reshaping. The reshaped 280 nm UV trace and the four EMG + GMG peaks are shown in the background (no scale given): mG-Pk1, green and olive squares; mG-Pk2, red and wine circles; mG-Pk3, magenta and purple triangles; mG-Pk4, blue and navy diamonds. The s.d. values associated with each point derive from the weighted linear regression analyses.

As can be seen in supplementary Fig. S8, without Gaussian decomposition the *R*_g_ value changes continuously from the first frames examined (when material starts eluting) up to the beginning of the main peak, indicating that more than one species contributes to each time frame up until about frame 135, owing to incomplete separation. In contrast, when the EMG + GMG decomposed data are analyzed, selecting a single Gaussian and the appropriate frame region separately, highly improved flat *R*_g_ *versus* frame plots result, as can be seen in supplementary Fig. S9 (see also Fig. 8 ▸), with the exception of peak mG-Pk1. This is due to the very likely presence of more than one species under this peak, as shown by the global decrease of *R*_g_ (and *M*_w_[RT], plot not shown) values with increasing frame number.

### 3.5. Decomposition and band-broadening correction for a concentration signal   

To derive a complementary molecular mass estimate from the *I*(0)/*c* value, a concentration monitor chromatogram can be likewise decomposed. It is important to point out that concentration-related data are uploaded and internally treated separately from the SAXS data, with which they are then associated. This concentration-related data set is first rescaled on a chosen *I_q_*(*t*) chromatogram to have both curves clearly visible (Brookes *et al.*, 2013 ▸). At most beamlines, concentration and SAXS detectors probe separate volumes downstream from the column. The concentration chromatogram must therefore be time-realigned with the SAXS chromatogram (Brookes *et al.*, 2013 ▸). The rescaled time-shifted concentration signal is then selected and fitted using the current set of Gaussians. Importantly, only a minimal variation in the Gaussian centers optimized on the SAXS data set is allowed (2% from initial values by default), leaving only widths, distortions and amplitudes to be optimized. However, widths and distortions are coupled parameters. From our recent experience, it appears to be more efficient at this step to keep the widths fixed (default) and let only distortions vary. The results of this procedure on a 280 nm UV trace collected on a diode-array detector (DAD) placed before the SAXS detector for the aldolase SE-HPLC-SAXS data examined here are shown in supplementary Fig. S10 (since most concentration-related data sets do not carry associated s.d. data, the residuals are shown on an absolute scale). Although the fitting could probably be improved by releasing some constraints, it is important to stress that a correspondence with the SAXS-optimized data was the primary goal. The concentration-related data set can now be associated with the *I_q_*(*t*) chromatograms.

The main reason for seeking a tight correspondence with the SAXS-derived data resides in an additional implemented feature, dealing with band broadening between the concentration and SAXS detectors. We now offer a re-shaping of the concentration Gaussians based on the SAXS-optimized Gaussians, while keeping each concentration Gaussian area constant. In other words, a concentration profile is recreated using the individual shapes of the SAXS Gaussians and the areas of the concentration Gaussians. When the *I_t_*(*q*) frames are back-generated, it is possible to enter values of extinction coefficients [or the d*n*/d*c*(s)] and partial specific volume(s) to be associated with each Gaussian. The concentration-related Gaussians are then used to associate a concentration value with each frame. When exported to the main *US-SOMO* SAS module and processed with the ‘Guinier’ utility, estimates of the molecular masses are thus derived directly from the intercepts of the linear regressions. With the reshaping option, a better correspondence between the concentrations and the SAXS intensities should be obtained. However, this procedure effectively results in molecular mass estimates whose variation along a given decomposed Gaussian peak just reflects the small departures from the Gaussian fit present in the SAXS data. The molecular mass thus can be thought of as artificially ‘constant’. The results of utilizing the non-reshaped and reshaped concentration Gaussians can be seen in supplementary Figs. S11 and S12, respectively, where a number of frames under each EMG + GMG peak have first been normalized by their associated concentrations and then plotted (an average curve of the normalized frames is also plotted). As can be seen, while the normalized curves obtained utilizing the reshaped concentration curves appear to superimpose well on top of each other (supplementary Fig. S12), this is not the case for the non-reshaped set (supplementary Fig. S11). The effectiveness of the reshaping was further confirmed by a scaling analysis (not shown), resulting in scaling coefficients of 1.0 within around 3% on average. Guinier plots of the normalized average *I_t_*(*q*) curves for the four aldolase EMG + GMG peaks are presented in supplementary Fig. S13, where the excellent quality of the resulting data sets can be appreciated.

The results of the Guinier analyses in terms of [〈*R*_g_^2^〉*_z_*]^1/2^ and 〈*M*〉_w_ values for all the selected *I_t_*(*q*) frames, normalized after concentration curve reshaping, are shown in graphical form in Fig. 8 ▸, superimposed on the reshaped 280 nm UV trace and its EMG + GMG components. A summary of the data is presented in Table 1 ▸, where an additional estimate of the 〈*M*〉_w_ values is also given that is independent of the sample concentration (column 7). This is obtained from the *SAXS-MoW* program (Fischer *et al.*, 2010 ▸; a newer version, *SAXS-MoW2*, is now available at http://www.if.sc.usp.br/) which is based on the determination of the Porod volume. As can be seen, very consistent data are obtained, with the largest variability (up to ∼5% in the [〈*R*_g_^2^〉*_z_*]^1/2^ values) observed for mG-Pk1, which probably regroups non-resolved oligomers and for which not enough low-*q* points were available (see also supplementary Fig. S9). As for the other peaks, the variability in the [〈*R*_g_^2^〉*_z_*]^1/2^ values is often below 1%. Furthermore, for these peaks very little variation exists between the values calculated from, respectively, the top *I_t_*(*q*) frame, the average frame or the means of the calculated values for each single frame, these last showing the largest s.d. values. Regarding the 〈*M*〉_w_ values, it is instructive to compare them with the values that can be computed from the rabbit muscle aldolase composition, which physiologically is a homotetramer of 157.131 kg mol^−1^ (Blom & Sygusch, 1997 ▸). As can be seen from columns 4 or 7 in Table 1 ▸, the Guinier region of peak mG-Pk4 gave a practically exact value, while the values obtained from the Guinier region of peaks mG-Pk3 and mG-Pk2 are within approximately −6 to −7% of those of a dimer and a trimer of homotetramers (octamers and dodecamers), respectively. These already quite satisfactory discrepancies are, however, two to three times larger than those observed using *SAXS-MoW* (2–2.5% discrepancies). They are likely to result, at least in part, from a still-not-perfect reshaping of the concentration signal, a difficult process. As for the values for peak mG-Pk1, they show more variability, the highest being very close (∼2%) to that of a hexamer of homotetramers.

### 3.6. Decomposed *I_t_*(*q*) *versus q* data sets can be used for molecular modeling   

**Figure 9:**
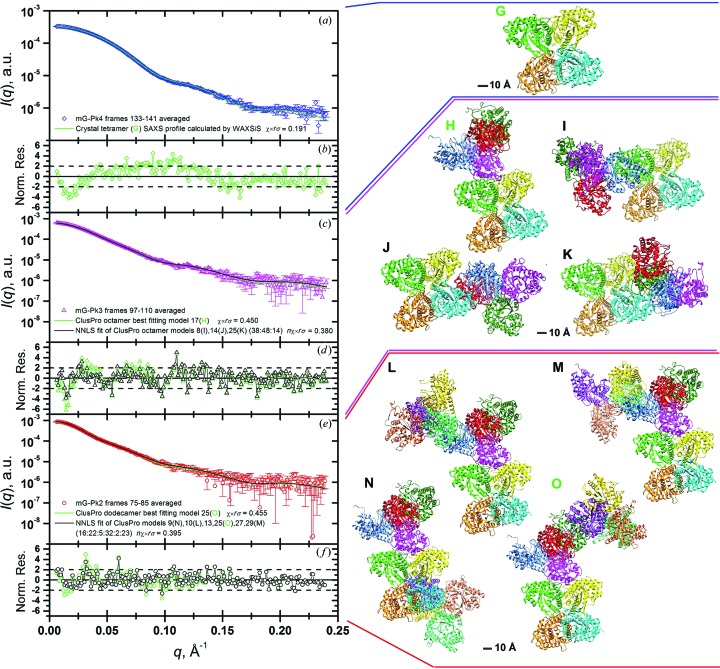
Fitting of model curves, based on crystallographic and docked structures, on the averaged frames for mG-Pk4 (panel A), mG-Pk3 (panel C) and mG-Pk2 (panel E). In panels B, D and F, the respective normalized fit residuals [(*I*(*q*)_fit_ − *I*(*q*)_expt_)/s.d._expt_] are reported (the dashed horizontal lines indicate the ±2 s.d. limits). The χ*r*_σ_ values [equation (12)] are also reported in the inside legends in panels A, C and E. (Panel G) The aldolase crystallographic tetramer (PDB code 1ado). (Panel H) The best mG-Pk3 SAXS single-fitting *ClusPro* aldolase octamer (model No. 17). (Panels I–K) *CluspPro* octamers Nos. 8, 14 and 25, respectively, whose 36:48:14% combination produces the overall best fit to the mG-Pk3 SAXS profile. (Panels L–O) The four *ClusPro* aldolase dodecamer models contributing most to the NNLS best reconstructed curve for the mG-Pk2 SAXS profile (L, No. 10, 22%; M, No. 29, 23%; N, No. 9, 16%; O, No. 25, 32%; model Nos. 13 and 27, not shown, contribute 5 and 2%, respectively). Model No. 25 (panel O) is the single best fitting model. In all panels, each aldolase monomer is colored differently, while to make a comparison easier the orientation of the starting tetramer (G) is the same in all higher-order complexes produced.

We can now compare the average top 11 or so *I_t_*(*q*) frames (adding more lower-intensity frames from both sides of the decomposed peaks did not improve the quality of the averaged frames) for the peaks corresponding to well defined species with those that can be computed from the aldolase crystal structure (see *Materials and methods*, §2.1). The biological unit extracted from the 1ado PDB file (Blom & Sygusch, 1997 ▸) is a homotetramer (Fig. 9 ▸, panel G) and, as can be seen in Fig. 9 ▸ (panels A and B), a quite satisfactory agreement between the computed *I*(*q*) curve and the average curve for frames 133–141 of peak mG-Pk4 is observed by scaling the two curves. The relatively minor discrepancies that are apparent in the residuals plot, especially in the Guinier region (*R*_g_ values of 36.0 and 35.6 Å from experimental and calculated patterns, respectively), are likely to depend on conformational variability existing at the C-terminal ends of the aldolase subunits (Blom & Sygusch, 1997 ▸), which will give rise to several conformers in solution. However, no attempt was made to improve the fit by exploring this possibility, since this was outside the scope of this work.

As for the higher-order complexes, their existence has been validated beyond reasonable doubt by the analysis of our SEC-SAXS data. Furthermore, the fact that individual peaks are present, albeit not fully resolved during elution through the SEC column, suggests that they are really distinct species, and not part of an equilibrium between the stable tetramers and their association into higher-order complexes. Since each rabbit muscle aldolase subunit has eight cysteines but no intra-chain disulfide bridges (Lai *et al.*, 1974 ▸; Blom & Sygusch, 1997 ▸), the formation of inter-chain S—S bridges was considered. However, both SDS-PAGE (sodium dodecyl sulfate polyacrylamide gel electrophoresis) analyses under non-reducing conditions and SE-HPLC runs in the presence of up to 20 m*M* dithiothreitol failed to support this hypothesis (data not shown). While fully determining the binding/bonding nature of these higher-order aldolase complexes was beyond the scope of this work, we nevertheless attempted to model their mutual arrangement. We have thus resorted to a docking program (*ClusPro 2.0*; see *Materials and methods*, §2.1), which allows for generating and then screening putative complexes also on the basis of the agreement of their internally calculated scattering patterns with an input SAXS curve. This procedure generated 30 ‘balanced’ (*i.e.* with comparable contributions from electrostatic, hydrophobic and van der Waals binding forces) models for the octamer (dimer of tetramers), for each of which the *I*(*q*) curve was then re-computed. Searches for the single best fitting curve, and for a combination of curves giving the best fit, were conducted against the average curve for frames 97–110 of the aldolase mG-Pk3, using the non-negative least-squares (NNLS) routine in the *US-SOMO* SAS module. As can be seen in Fig. 9 ▸ (panels C and D), remarkably good fits were obtained, with model No. 17 (Fig. 9 ▸, panel H) being the single best fitting curve, and a combination of model Nos. 8, 14 and 25 (Fig. 9 ▸, panels I–K) giving a slightly improved score [normalized χ multiplied by *r*_σ_; equation (12), see *Theory*, §2.3.3]. Starting from the best octamer model found in the previous step, *ClusPro 2.0* was again used to find putative dodecamers (trimers of tetramers), using the HPLC-SAXS averaged frames 75–85 for the mG-Pk2 curve as a constraint. SAXS profiles were then re-computed for the resulting 30 balanced models. As can be seen in Fig. 9 ▸ (panels E and F), excellent NNLS fits could again be obtained for either a single best fitting model (model No. 25; Fig. 9 ▸, panel O) or a combination of several models (Nos. 9, 10, 13, 25, 27 and 29 in a 16:22:5:32:2:23% ratio; only model Nos. 10, 29 and 9 are additionally shown in Fig. 9 ▸, panels L, M and N, respectively).

## 4. Discussion   

We have presented a vastly improved version of a data-analysis module specifically developed for processing real-life SEC-SAXS data. Beyond the case of well resolved symmetric elution peaks, it offers solutions to handle severe capillary fouling issues, as well as asymmetric and poorly resolved peaks that are frequently encountered. The protocol developed for baseline correction following capillary fouling is model dependent and would probably not apply if a very different fouling mechanism were at work. However, we consider the proposed algorithm to be physically plausible and we have shown it to be effective in a particularly severe case. In addition, band-broadening issues when using separated concentration and SAXS detectors can be significantly atten­uated by reshaping the concentration signal on the experimental SAXS profile. Advanced statistical tools are now available to validate operations/results and to guide the user’s choices at each step. The ability of our approach to retrieve structural information from a SEC-SAXS data set comes at the price of extra complexity. For the time being, it is far from being automated and cannot be considered as a high-throughput tool, although we contemplate automating several steps in a future release.

The major improvement in sample quality offered by SEC-SAXS explains its availability at a growing number of synchrotron radiation facilities worldwide and the correlated developments of specific software. For instance, *DATASW* (Shkumatov & Strelkov, 2015 ▸) provides a user-friendly interface for identical frame averaging and publication-quality figure preparation, but does not venture much further. Furthermore, a recent report describes an automated pipeline for the SEC-SAXS setup available at the EMBL-P12 beamline at PETRA 3, Hamburg (Graewert *et al.*, 2015 ▸). The major original feature of the setup is that, thanks to a micro-splitter valve, it allows the parallel monitoring of the eluted solution by SAXS and by a triple detector array (UV-absorption, light scattering and refractive index), a very interesting approach. However, no attempt is presented to decompose the elution profile into the various contributions from the eluting species. Finally, a recent article presents novel methods for the analysis of SEC-SAXS data (Malaby *et al.*, 2015 ▸). These methods are based on SVD and so-called Guinier-optimized linear combination to facilitate data analysis and reconstruction of protein scattering directly from peak regions. While the use of SVD for a refined buffer subtraction is of great interest, the reconstruction aspect is more limited and does not lead to a complete decomposition of the SEC-SAXS data sets into individual species contributions.

As already mentioned in the introduction, the HPLC-SAXS module within *US-SOMO* also offers SVD analysis, used to determine in an unbiased way the minimum number of species accounting for the entire data set. This guides the subsequent choice of (modified) Gaussians [(m)G-Pk(*i*)] used in the decomposition process. However, we also wondered if SVD could be put to more efficient use. Indeed, once the choice of the number of species *N*_sp_ is determined, all other singular values (and associated vectors) represent noise in the data. It is thus legitimate to use, instead of the noisy original SAXS frames, their projection into the subspace of the first *N*_sp_ singular vectors, thereby filtering out much of the experimental noise of individual frames. We performed a parallel analysis of the aldolase data set using the projection of experimental frames onto the first four singular vectors, in the hope of being able to extend our data to higher *q* values and improve the consistency of the reconstructed curves. Although the projected patterns were much less noisy, the corresponding gain for the reconstructed curves was much smaller. Further work is required before drawing a definitive conclusion on the interest of a preliminary SVD filter.

However, we reasoned *a posteriori* that our (modified) Gaussians were determined through a global fit and that this operation implicitly performed a filtering function similar to that carried out by SVD. While both methods determine the basis set of functions used for the decomposition by minimizing the global mean-square discrepancy between experimental frames and their reconstructions, a major difference regards the way they deal with the time dimension of the data set. SVD simply ignores it. Indeed, the singular values and singular vectors are absolutely independent of the time sequence of the scattering patterns. In contrast, our decomposition of the data set using a small number of (modified) Gaussians relies entirely on the time profiles of the scattered intensities. The incorporation of this essential time information is at the heart of the method and explains why we are able to restore actual scattering profiles and not only a set of basis vectors that, except for the first singular vector, are not scattering curves. This decomposition relies on physically meaningful modeling of the elution process of molecules along the SEC column.

The introduction of a routine implementing the *CorMap* approach recently proposed by Franke *et al.* (2015 ▸) to evaluate the similarity between scattering curves (or chromatograms) constitutes a major help in the decision-making and results evaluation that are now available. It complements beautifully the χ^2^ statistics that depend fully on the accuracy of the uncertainty estimates. This is clearly visible in supplementary Fig. S6 (bottom frame), showing the distribution of both *P* values and χ^2^ values as a function of *q* obtained from the pairwise comparison of each chromatogram and its fit using the four modified Gaussians. The two distributions are very different. The results exhibit low (and high) *P* values distributed over the entire *q* range. In contrast, the χ^2^ values follow a well defined *q* dependence, with a peak between 0.02 and 0.07 Å^−1^. This is more a reflection of the *q* dependence of the magnitude of the experimental uncertainties than of actual variations in the quality of the fit. Indeed, at the SWING beamline, uncertainties are derived from intensities assuming Poisson statistics and no systematic bias from the detector is taken into account. What the comparison of the two profiles reveals is that, in regions of *q* where the ratio of counting statistics over intensity is largest, this systematic detector bias is no longer negligible. Finally, the stringency of the test when comparing scattering curves can be modulated by adjusting the *q* range taken into consideration, mostly by focusing on the small-angle region. Indeed, we perform, at times in parallel, a twofold *P*-value analysis, one over the entire useful *q* range and another one restricted to *q* values lower than 0.05 Å^−1^ to improve the detection of systematic differences at low *q* that might have gone unnoticed. The matter of test stringency is made more complex by the issue of multiple testing effects and the ways to correct for it. Although a clear improvement over the simple Bonferroni procedure, the Holm–Bonferroni adjustment appears, at least in our case, to be prone to type II errors, considered as equal curves that exhibit genuine differences. This is illustrated by the results of the analysis of buffer frames using all *q* values shown in supplementary Fig. S2, in which 99.8% of all pair comparisons yield *P* values deemed acceptable after Holm–Bonferroni adjustment, while the analysis of the same data without it makes clear the existence of correlations between adjacent *q* values (supplementary Fig. S1). Therefore, we offer the Holm–Bonferroni adjustment as a routine tool but suggest performing the comparative uncorrected analysis in case of doubt, *i.e.* if the HB-adjusted map of pairwise *P* values is not uniformly green.

That most frames in a SEC-SAXS data set correspond to a mixture and not a monodisperse solution results directly from the comparison between an experimental frame, its fit by the combination of (modified) Gaussians and the individual Gaussians (see Fig. 7 ▸). While frames on the right-hand side of the aldolase tetramer peak only contain contributions from mG-Pk4, not a single frame reduces to a unique contribution in the frame range 55–135. Most striking is the case of mG-Pk2, in which each experimental frame contains a very significant contribution from mG-Pk1 or mG-Pk3. In spite of the very poor resolution, our decomposition protocol leads to reconstructed curves for the various peaks that are, with the exception of mG-Pk1, highly self-consistent [a very small dis­persion between the reconstructions 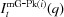 over the various frames]. mG-Pk1 is a special case, since the reconstructed profiles exhibit a systematic evolution with time (see for instance the [〈*R*_g_^2^〉*_z_*]^1/2^ and 〈*M*〉_w_ values in Fig. 8 ▸), strongly suggesting that this peak actually regroups an unresolved mixture of oligomers from the hexamer of tetramers [as illustrated by the molecular mass value derived from the highest *I*(0) value] to the tetramer of tetramers. In contrast, the other three peak scattering patterns yield molecular masses very close to those of a tetramer, and to a dimer and a trimer of tetramers, respectively (see Table 1 ▸). Furthermore, the scattering pattern calculated from the complete aldolase crystal structure is very similar to the curve of the tetramer peak (see Fig. 9 ▸, panels A and B). Finally, using the program *ClusPro2.0* with SAXS restraints we could build dimers and trimers of tetramers, the scattering patterns of which were already close to the corresponding peak curves, their combination providing even better fits. The reconstructed curves for both peaks mG-Pk2 and mG-Pk3 are thus perfectly compatible with *bona fide* oligomers of the tetramer. Our protocol therefore appears capable of recovering from a data set of essentially mixtures of oligomers the scattering patterns of isolated components. It is also worth noting that the consistency of both protocols can be checked internally simply by comparing scaled curves from a single peak of baseline-corrected data. We believe that this decomposition procedure, together with the integral baseline-correction routine, allows the experimentalist who collected the SEC-SAXS data to extract most of the structural information content of the data set into reliable profiles of purified species for further characterization and modeling using tools developed for monodisperse samples.

## Supplementary Material

Additional material and figures. DOI: 10.1107/S1600576716011201/vg5038sup1.pdf

## Figures and Tables

**Table 1:** Summary of the Guinier (columns 3–6) and *SAXS-MoW* (column 7) analyses of the EMG + GMG decomposed aldolase HPLC-SAXS data

EMG + GMG peak No.	Frame(s)	[〈*R_g_* ^2^〉*_z_*]^1/2^ (Å)	Guinier 〈*M*〉_w_ (kg mol^−1^)	*q*_min_ − *q*_max_ (Å^−1^)	χ^2^	*SAXS-MoW* 〈*M*〉_w_ (kg mol^−1^)
1	60 (top)	101.1 ± 2.8	925 ± 22	0.00743–0.01257	0.0205	n.d.
1	Average of frames 53–72 results	94.4 ± 5.1	886 ± 40	n.a.	n.a.	n.d.
1	Average frame of frames 53–72	94.8 ± 1.4	897 ± 11	0.00743–0.01372	0.0126	1000 ± 3
2	80 (top)	63.6 ± 0.9	441 ± 4	0.00800–0.01886	0.0152	n.d.
2	Average of frames 67–94 results	63.1 ± 1.7	432 ± 10	n.a.	n.a.	n.d.
2	Average frame of frames 67–94	63.4 ± 0.3	434 ± 1	0.00800–0.01886	0.0058	462 ± 1
3	102 (top)	53.5 ± 0.6	297 ± 2	0.00857–0.02229	0.0127	n.d.
3	Average of frames 90–113 results	52.1 ± 1.4	292 ± 5	n.a.	n.a.	n.d.
3	Average frame of frames 90–113	52.0 ± 0.3	292 ± 1	0.00857–0.02286	0.0065	306 ± 4
4	137 (top)	36.4 ± 0.1	156 ± 0	0.00857–0.03143	0.0015	
4	Average of frames 126–150 results	36.1 ± 0.4	157 ± 2	n.a.	n.a.	n.d.
4	Average frame of frames 126–150	36.0 ± 0.1	157 ± 0	0.00857–0.03143	0.0031	154 ± 1
