# Supplementary material for: US-SOMO HPLC-SAXS module: dealing with capillary fouling and extraction of pure component patterns from poorly resolved SEC-SAXS data
Source: J Appl Crystallogr. 2016 Sep 1;49(Pt 5):1827–41. doi: 10.1107/S1600576716011201 (PMC5045733; doi:10.1107/S1600576716011201)
Supplement: Supplementary file 1 [file j-49-01827-sup1.pdf]

JOURNAL OF APPLIED CRYSTALLOGRAPHY

Volume 49 (2016)

Supporting information for article:

US-SOMO HPLC-SAXS module: dealing with capillary fouling, and extraction of pure component patterns from poorly resolved SEC-SAXS data

Emre Brookes, Patrice Vachette, Mattia Rocco and Javier Pérez

S1. Additional program features.

The data shown in Fig. 1 (main text) were semi-automatically trimmed by a new optional function introduced in this release (this is useful to remove data with no pertinent information, and should be applied in most cases). This function is meant to remove from further analysis the $I_q(t)$ curves carrying only noise. A test is made to detect at least one point in each produced $I_q(t)$ *vs.* t curve where the $I_q(t)$ value is greater than the experimental standard deviation (SD) associated to that point multiplied by a chosen value (3 by default). Any $I_q(t)$ *vs* t curve that fails the test is assumed to contain no useful signal and is dropped; a message appears in a pop-up box listing the first 15 instances, sorted by increasing q-values, of such an occurrence and signaling how many more were found. Most of these occurrences will correspond to the higher q-range where the signal is weakest. This simple test provides a rough estimate of the maximal useful q-value and accordingly reduces the data set. In addition, a check is performed to detect suspicious regions in the $I_q(t)$ curves with a large range of negative values. This might occur at high q-values where the detector instabilities surpass the statistic errors, but it could also be indicative of less than ideal buffer (blank) subtraction. The occurrence of such regions of negative values could cause problems with the integral baseline subtraction. A sliding window (user-selectable width, here 25 frames) is used to identify regions where the sum of the intensities is less than the negative of the sum of the corresponding SD values over the window. If such regions are detected, the $I_q(t)$ curves will be identified and a warning issued.

S2. Holm-Bonferroni adjustment for multiple testing effects.

The description of the method is extracted from the corresponding Wikipedia article (https://en.wikipedia.org/wiki/Holm-Bonferroni_method).
Briefly, the method is as follows:

1-Let $H_1, \ldots, H_m$ be a family of m hypotheses and $P_1, \ldots, P_m$ the corresponding P-values
2-Start by ordering the P-values and their associated $H_1 \ldots H_m$ from lowest to highest
3-For a given significance level α, let k be the minimal index such that $P_k > \frac{\alpha}{m+1-k}$
4-Reject the null hypotheses $H_1 \ldots H_{(k-1)}$ and do not reject $H_k \ldots H_m$
5-If $k = 1$ then do not reject any of the null hypotheses and if no such k exist then reject all of the null hypotheses.

Here H_i regards the similarity of a pair of curves and P_i is the calculated P-value. In the final P-value map (see Fig. S2), the accepted hypotheses $H_k \ldots H_m$ (the two compared curves are identical) are represented in two colors corresponding to two values of α: yellow dots for $\alpha = 0.01$ and green dots for $\alpha = 0.05$, while $H_1 \ldots H_{(k-1)}$ for $\alpha = 0.01$ are represented as red dots (significant differences between the two curves).

S3. Additional Results Figures.

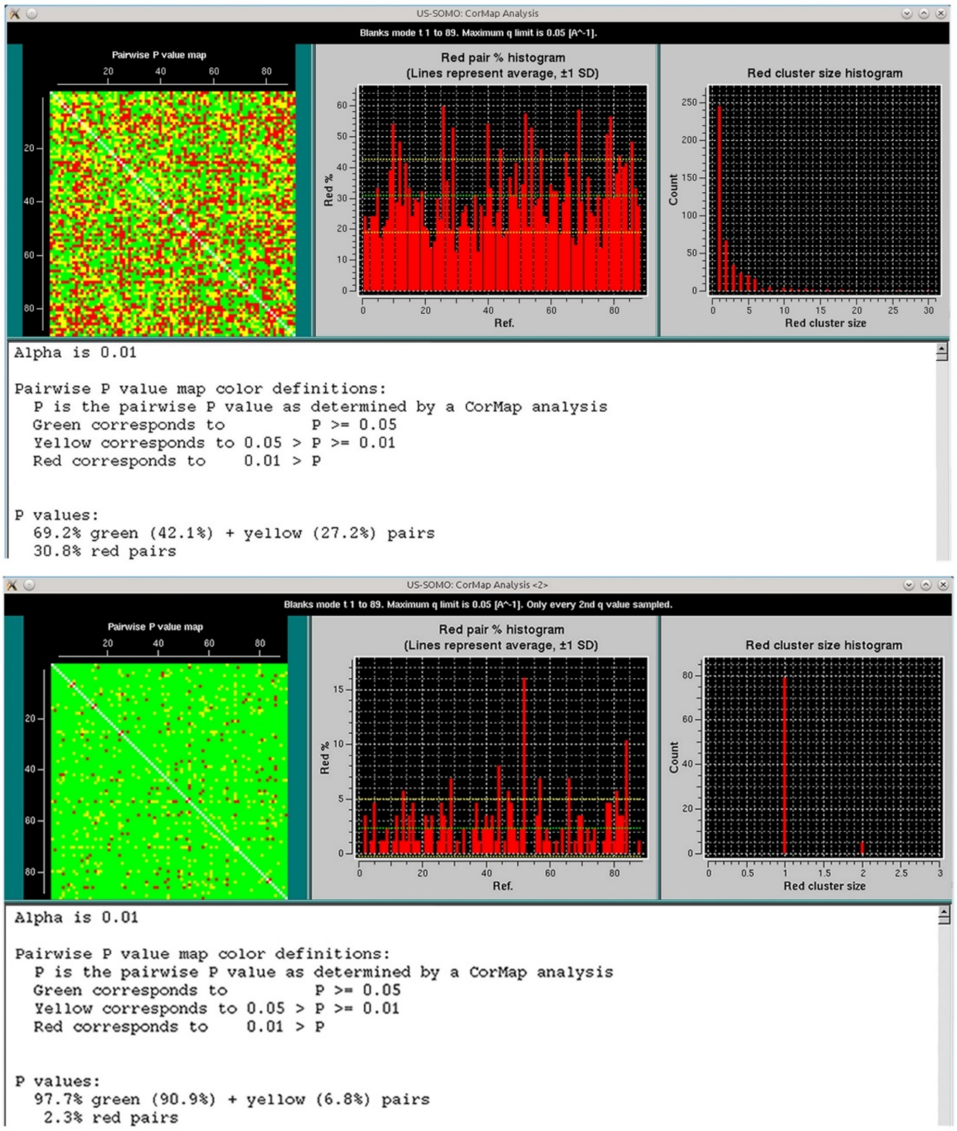

Figure S1 Pairwise *P*-value analysis without Holm-Bonferroni adjustment results of 89 buffer frames collected well before the SEC column void volume. Top image, using all *q*-values. Bottom image, using one every other *q*-value. A reference number (Ref.) is assigned to the first member of the pair of curves compared, and the correspondence with actual time/frame number is reported in the text area (not shown). In each image, the graphs include a novel pairwise *P*-value map, where each colored square represent the comparison between two data sets (see the text area for the color/*P*-value correspondence), a map of the fraction of red points as a function of Ref. number, and a Red cluster size histogram. In the text window, a summary of the analysis results is first given, followed by a detailed list first of the average results for each data set, and then of each pairwise comparison (only the *P*-values definition and the global results are shown here). All these data can be conveniently saved in csv format, and if necessary retrieved, using the "Save" and "Load" buttons.

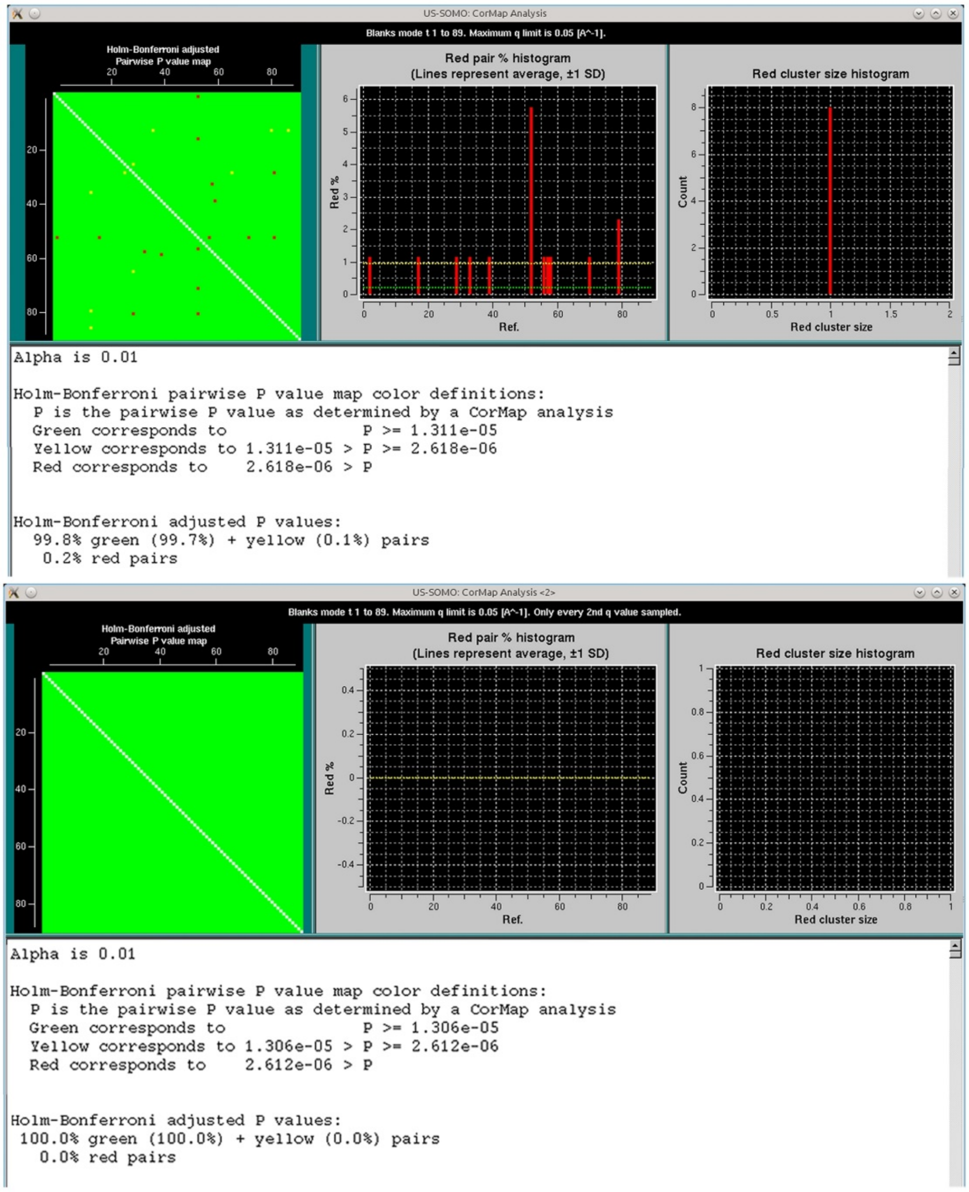

Figure S2 Pairwise *P*-value analysis with Holm-Bonferroni adjustment results of 89 buffer frames collected well before the SEC column void volume. Top image, using all *q*-values. Bottom image, using one every other *q*-value. See Fig. S1 legend for details.

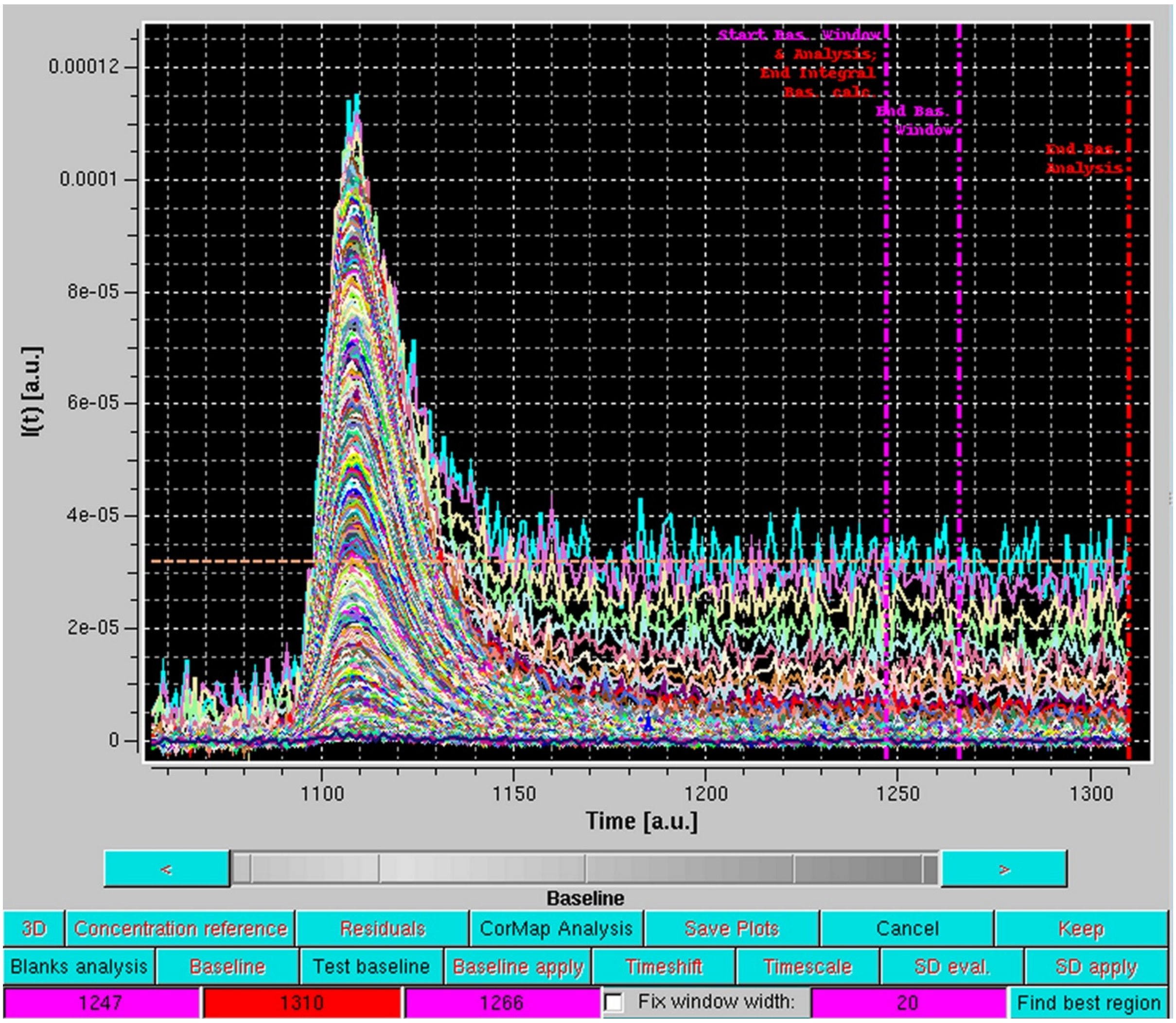

Figure S3 The Baseline mode of the US-SOMO HPLC-SAXS module. The leftmost magenta vertical line defines the beginning of the analysis window, and the sliding window analysis starting position (it will also define, once the analysis is completed, the position at which the integral baseline calculations will end). The rightmost magenta line defines the end of the analysis window. The vertical red line defines the end of the sliding window analysis. The horizontal orange line shows the average intensity of the lowest q-value data set over the region defined by the two magenta lines.

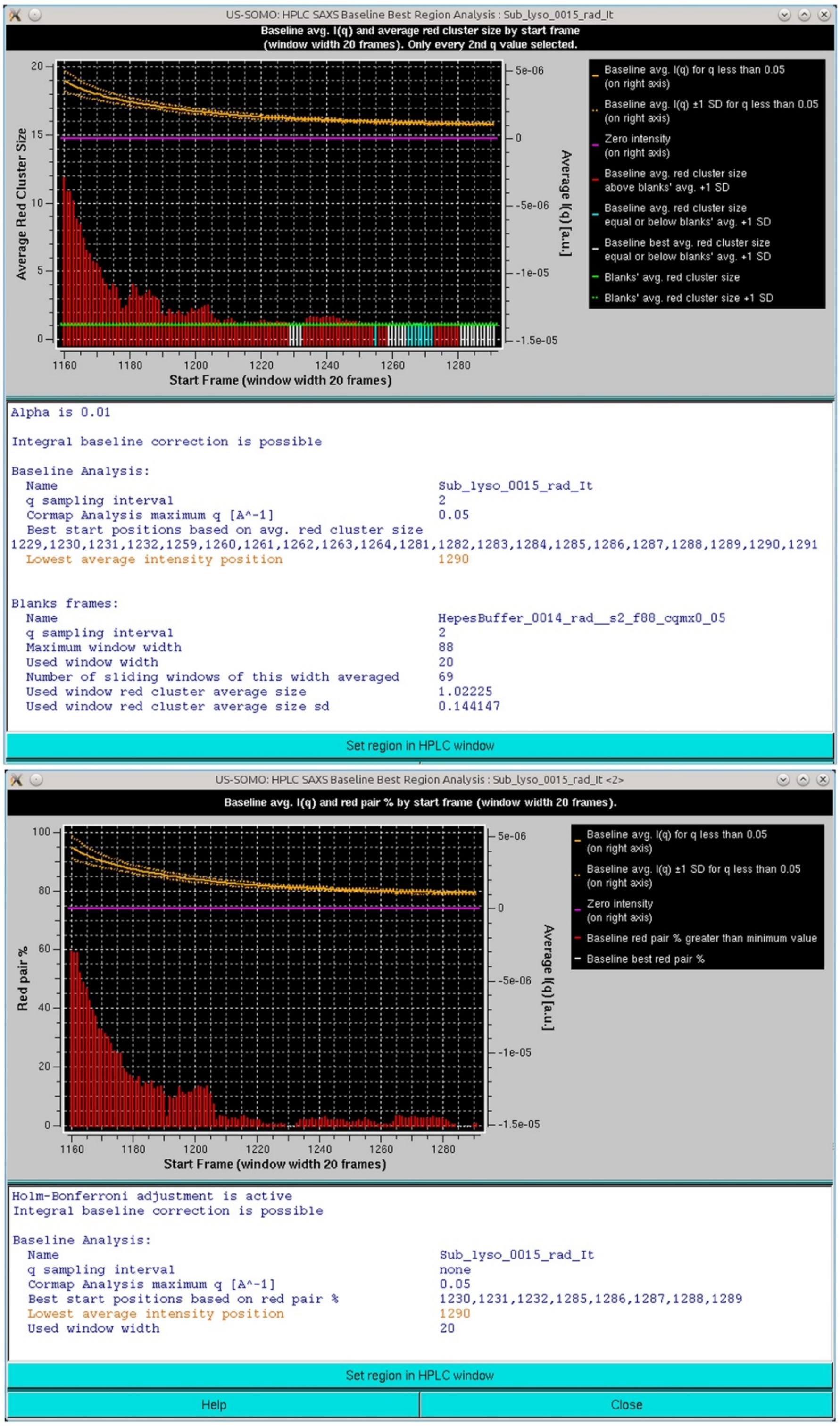

Figure S4 The actual pop-up panels for the "Find best region" in the US-SOMO Baseline utility. Top panel, results after the comparison with previously analyzed blanks and no Holm-Bonferroni adjustment. A sampling of one-every-other *q*-point was employed. Bottom panel, the same data analyzed without Blanks comparison, without sampling, and with the Holm-Bonferroni adjustment. In both panels the best starting frames positions for the integral baseline correction are reported, together with the lowest average intensity position.

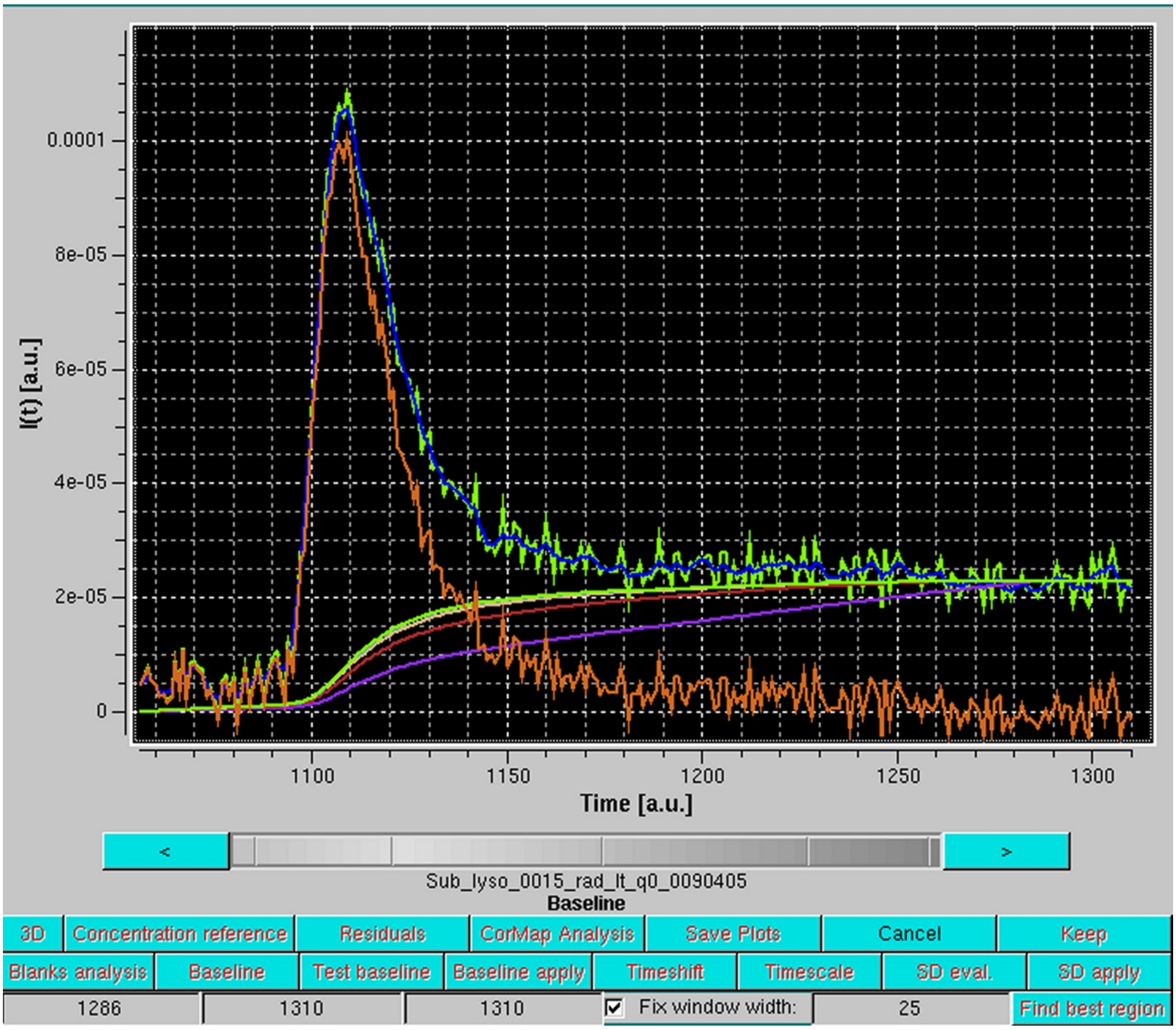

Figure S5 Testing the integral baseline correction on a $I_q(t)$ *vs.* t chromatogram. Green, original chromatogram at q = 0.0090 Å^{-1}; blue, the same chromatogram after a Gaussian smoothing with a width of 7; dark orange, the same original chromatogram after integral baseline correction; purple to light green lines, five subsequent iterations in the integral baseline definition (the fourth not being visible as it is completely superimposed by the fifth).

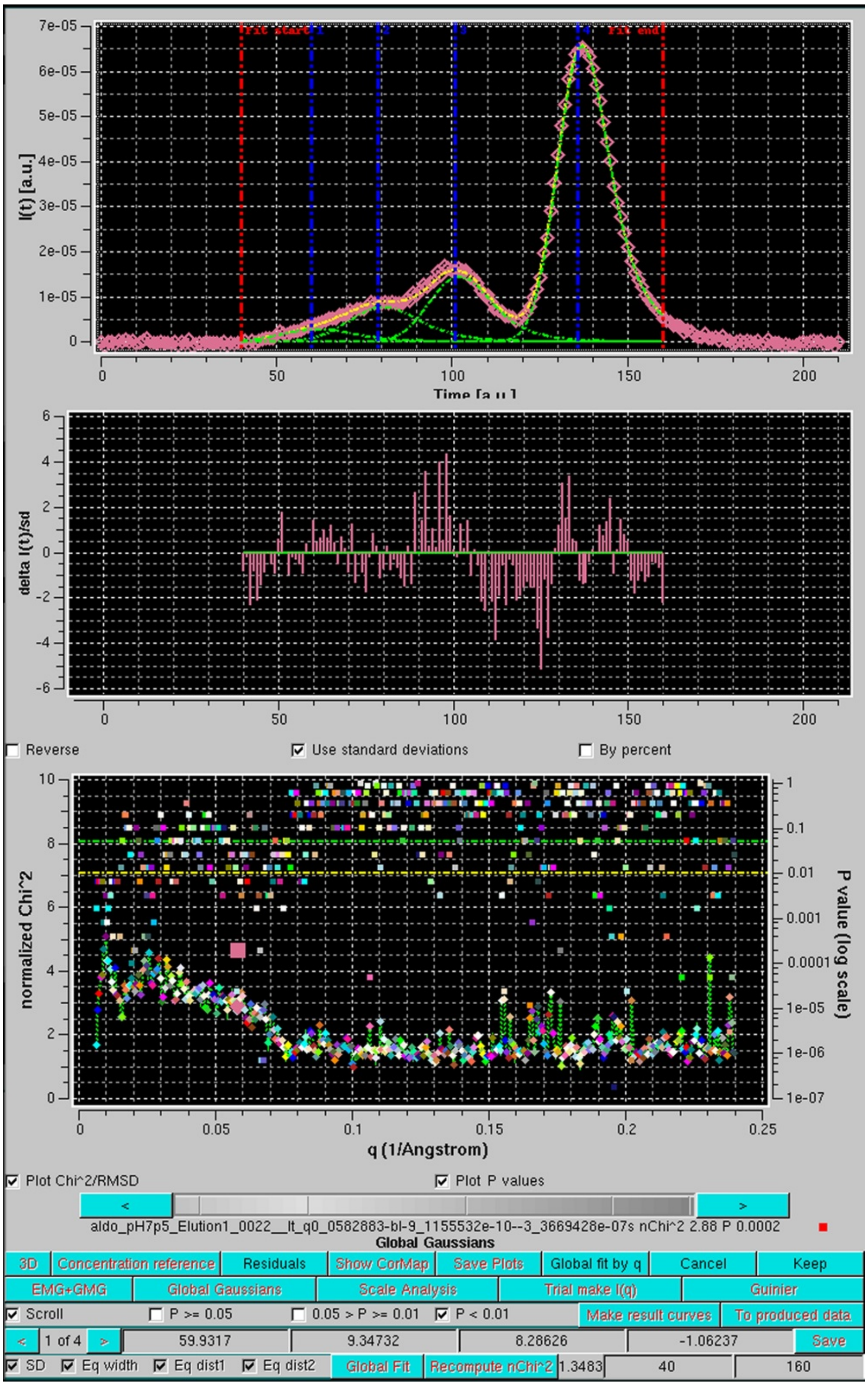

Figure S6 Top panel, original (points with error bars, pink diamonds) and reconstructed after EMG + GMG Gaussian decomposition (yellow line) $I_q(t)$ *vs.* t chromatograms for q = 0.05829 Å^{-1}, with the individual EMG + GMG Gaussians shown as green lines; the red vertical lines indicate the limits for the goodness-of-fit evaluation. Middle panel, the reduced residuals. Bottom panel, the “Global fit by q” plot, with both the normalized χ^2 (left y-axis, diamond connected by a line) and the pairwise P-values (right y-axis, squares) shown as a function of q; the currently scroll-selected chromatogram is indicated by the enlarged symbol. The horizontal yellow and green lines indicate the cut-off values for 0.05 > $P \geq 0.01$ and for $P \geq 0.05$, respectively.

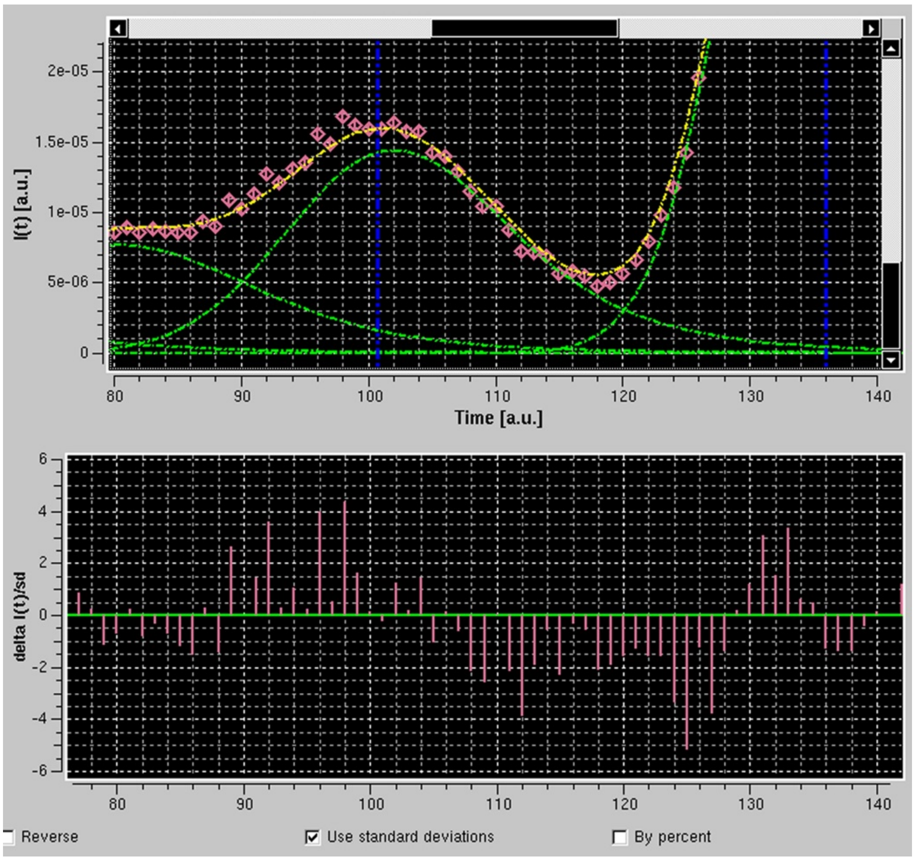

Figure S7 A zoom of the region having the longest stretch of consecutive SD values of the same sign for the $I_q(t)$ *vs.* t chromatograms at q = 0.05829 Å^{-1} shown in Fig. S6. Top panel, chromatograms. Bottom panel, associated reduced residuals.

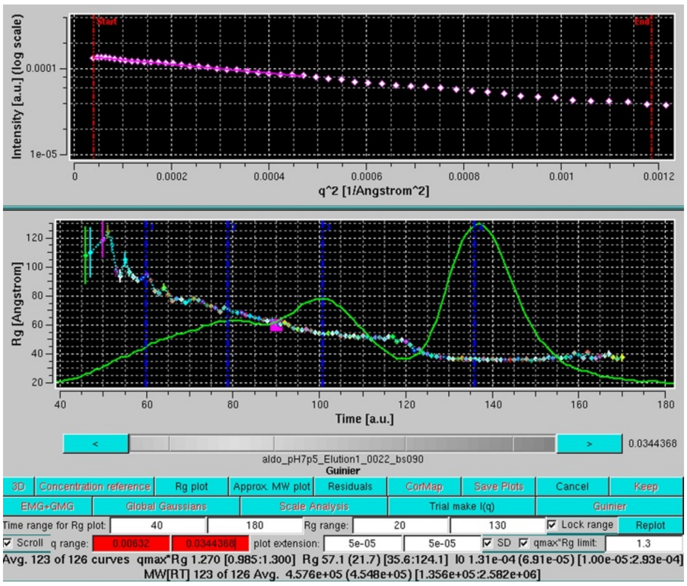

Figure S8 Guinier plot and R_g values before Gaussian decomposition. The Guinier plot, ln[$I_t(q)$)] *vs.* q^2, for a selected time point taken from the aldolase SE-HPLC-SAXS data without Gaussian decomposition is shown in the top panel (solid symbols, points included in the SD-weighted linear regression, automatically trimmed so to obey the "q_{max}*R_g" limit). Scrolling through the entire data set by using the grey-shades bar-wheel is made possible by enabling the "Scroll" checkbox. The computed R_g values are plotted as a function of the time/frame in the bottom panel, with a typical $I_q(t)$ *vs. t* chromatogram superimposed. The scroll-selected time point is indicated by an enlarged symbol (magenta in this case, as the plot in the Guinier panel).

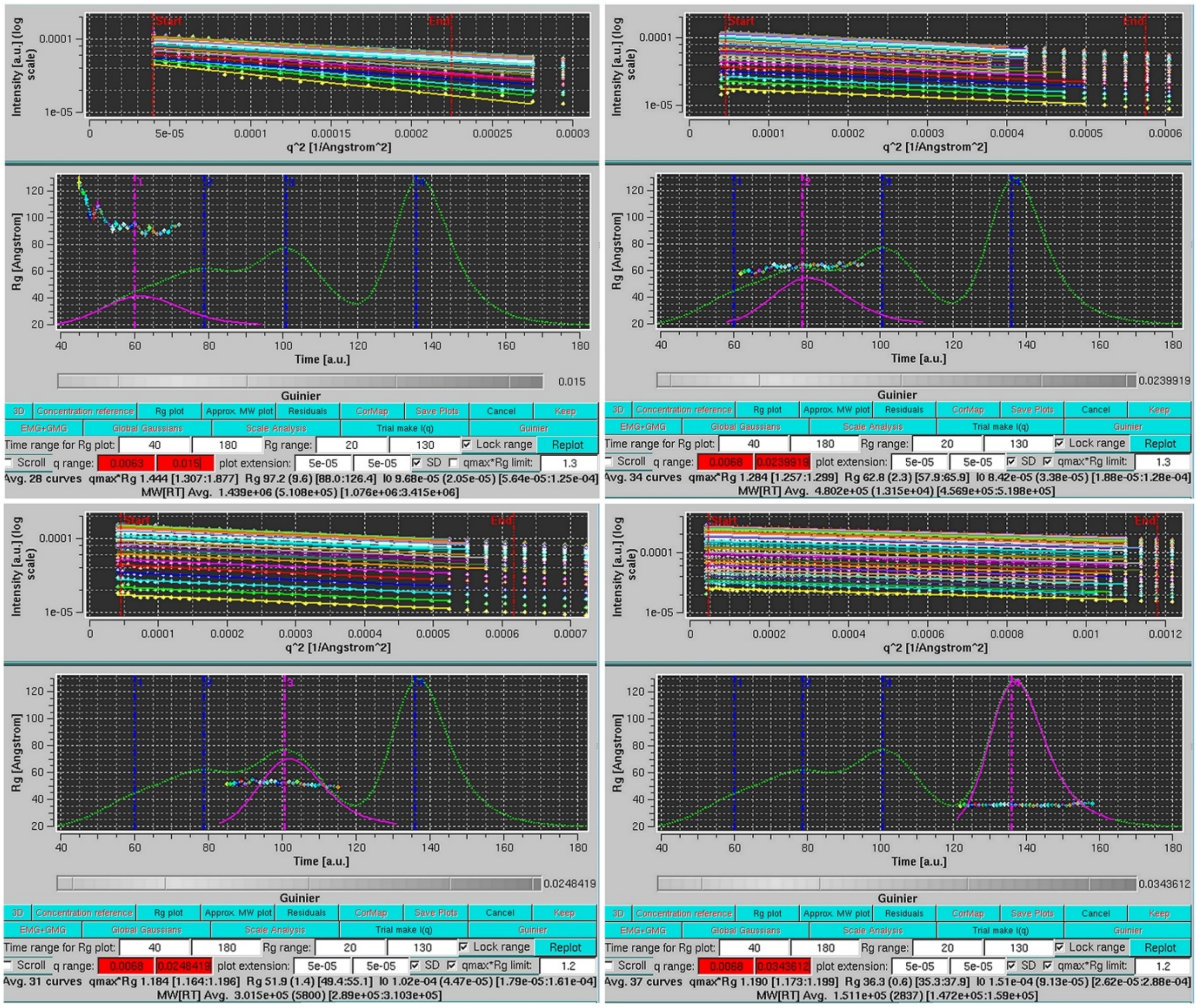

Figure S9 Composite screenshots from the US-SOMO HPLC-SAXS "Test I(q)" panel showing the Guinier plots for each of the four EMG + GMG components used in the decomposition of the aldolase SE-HPLC-SAXS data set (peak #1, top left; peak #2, top right; peak #3, bottom left; peak #4, bottom right) together with their associated R_g plots.

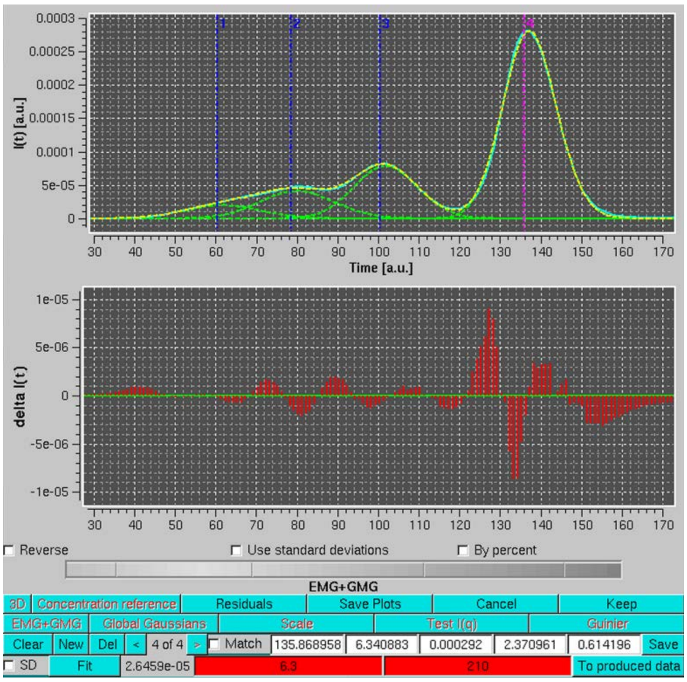

Figure S10 EMG + GMG fitting results of a 280 nm UV trace collected by a Diode Array Detector placed before the SAXS detector in the same SE-HPLC-SAXS aldolase run of Figs. 6-7 and S6-S9.

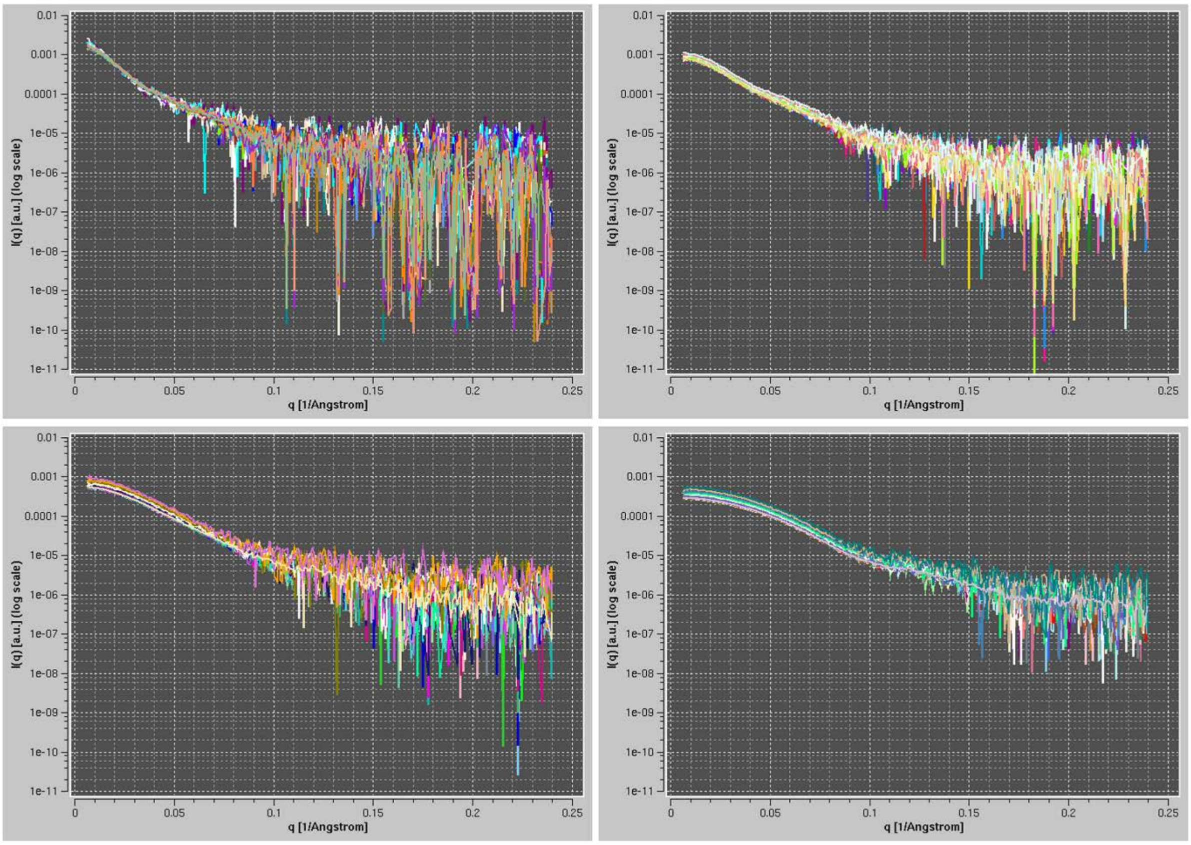

Figure S11 Superposition of the concentration-normalized, back-generated $I_t(q)$ *vs.* q frames for the EMG + GMG peaks #1 (top left, frames 48-72), #2 (top right, frames 67-94), #3 (bottom left, frames 90-116), and #4 (bottom right, frames 126-150), for the aldolase SE-HPLC-SAXS run described in the main text, without reshaping of the concentration chromatogram Gaussian peaks. In addition, the average of all frames is also plotted on top of the data in each panel (peak #1, olive; peak #2, cream; peak #3, pale yellow; peak #4, lilac).

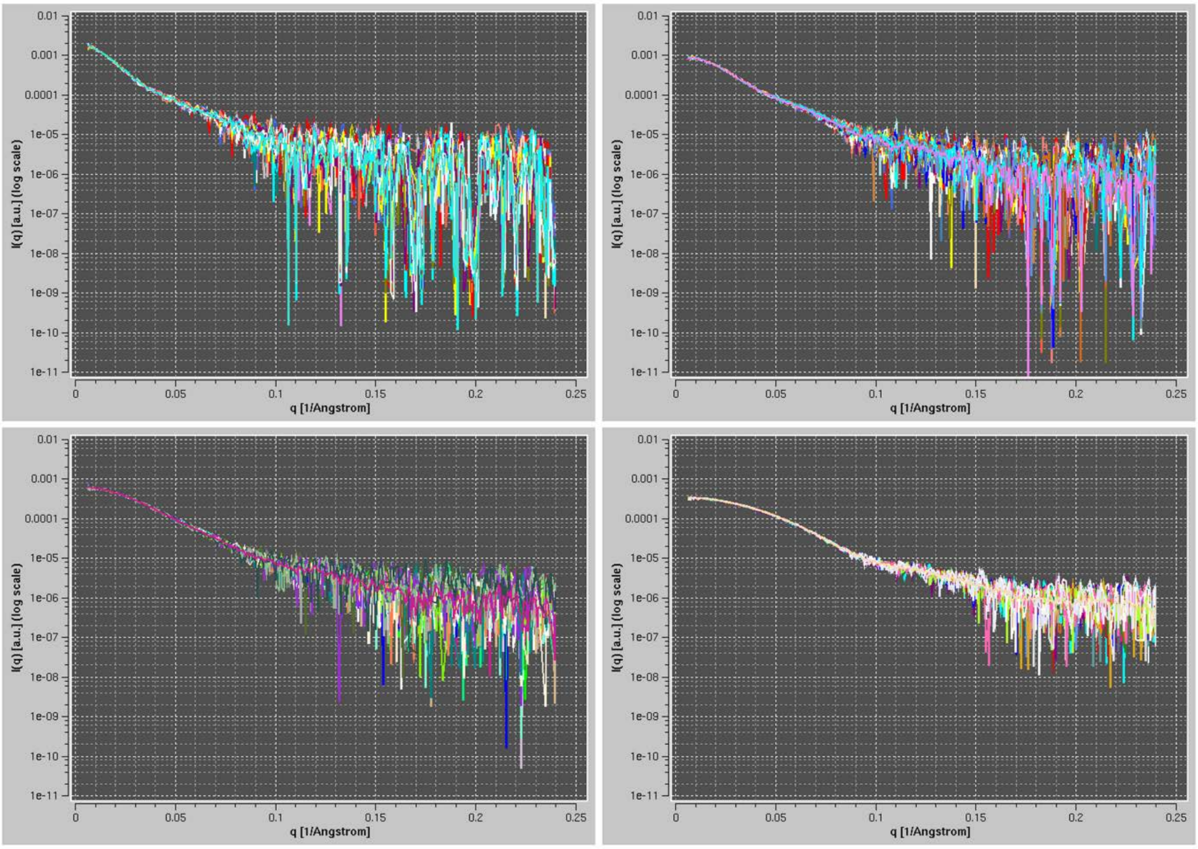

Figure S12 Effect of concentration chromatogram Gaussian peaks reshaping. Superposition of the concentration-normalized, back-generated $I_t(q)$ *vs.* q frames for the aldolase SE-HPLC-SAXS run described in the main text, for the EMG + GMG peaks #1 (top left, frames 48-72), #2 (top right, frames 67-94), #3 (bottom left, frames 90-116), and #4 (bottom right, frames 126-150). In addition, the average of all frames is also plotted on top of the data in each panel (peak #1, cyan; peak #2, purple; peak #3, magenta; peak #4, kaki).

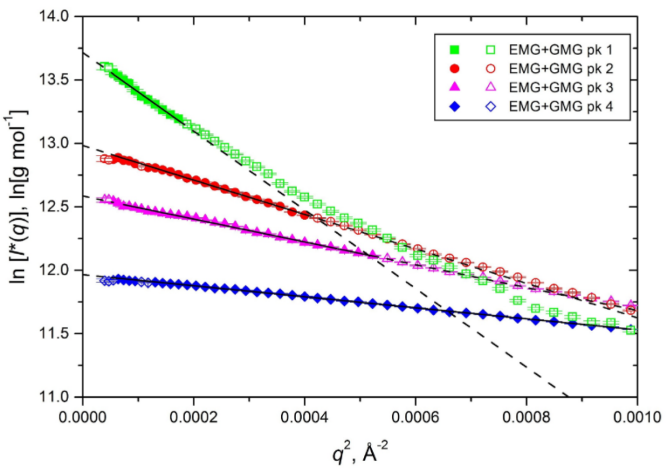

Figure S13 Guinier plots of the reduced $I_t(q)$ average frames for the EMG + GMG decomposed peaks of the aldolase SE-HPLC-SAXS analysis. Green squares, peak #1; red circles, peak #2; magenta triangles, peak #3; blue diamonds, peak #4. The black lines represent weighted linear regressions of the data, with the solid portion and the solid symbols defining the points included in the regression.
